# Optimising TB investments in Belarus, Moldova, Kyrgyz Republic, Tajikistan and Uzbekistan: An allocative efficiency analysis

**DOI:** 10.1371/journal.pgph.0004548

**Published:** 2025-07-11

**Authors:** Anna L. Bowring, Rowan Martin-Hughes, Debra ten Brink, Kelvin Burke, Svitlana Nidzvetska, Nisaa Wulan, Phillip Luong, Eloisa Perez-Bennetts, Nick Scott

**Affiliations:** 1 Burnet Institute, Melbourne, Australia; 2 Global Fund, Geneva, Switzerland; Heidelberg University, GERMANY

## Abstract

High rates of drug-resistant tuberculosis (TB) are a barrier to achieving End TB-strategy targets in Eastern Europe and Central Asia. This analysis collates results from five country-level modelling studies to identify priorities to reduce TB burden. Allocative efficiency studies were conducted in 2023 in Belarus, Kyrgyz Republic, Moldova, Tajikistan and Uzbekistan using the Optima TB model to determine the optimised distribution of funds to maximise health outcomes with given resources. A baseline scenario of continued 2022 spending was compared to scenarios with spending optimised across prevention, screening and treatment interventions to reduce TB incidence and deaths over 2024–2030. Modelled pulmonary TB incidence ranged from 25-119 per 100,000 population, and 14 − 43% of new/relapse TB cases were drug resistant. In all countries, optimizing current spending involved: expanding shorter treatment regimens (6–9 months) for drug-resistant-TB over standard regimens (18–20 months); reducing mass screening and mandatory testing and expanding community-based active case finding focused among populations at higher TB risk; and scaling-up TB preventive treatment. It was recommended to expand contact tracing in three countries and to improve cost-effectiveness in two countries by focusing on child household contacts first. With current spending optimised, pulmonary TB incidence was projected to decrease to 19 − 95 per 100,000 population by 2030, averting 1 − 13% of new/relapse TB cases and 1 − 18% of TB-related deaths from 2024-2030 compared to continued baseline spending. In three countries, optimised allocation of 150% of current spending had minimal additional epidemic impact. There are opportunities to reallocate TB funds more cost-effectively in Eastern Europe and Central Asia, but End TB targets may remain out of reach without new and prospective interventions.

## Introduction

Despite overall declines in global incidence of tuberculosis (TB), drug-resistant TB remains a major public health concern [1]. Ambitious targets set by the global End TB strategy aim for an 80% reduction in incidence rate and a 90% reduction in TB deaths by 2030 relative to 2015 [2]. However, many countries will face challenges in reaching End TB milestones, particularly following COVID-19-related disruptions in TB services and care-seeking which in 2020 reversed progress in many settings [1,3]. Further, drug resistance is an ongoing problem for TB control, with drug-resistant TB being more difficult to treat, requiring more costly and lengthy treatment, and having poorer treatment outcomes [1,4]. Subsequently, reducing drug-resistant TB is a key priority for both public health and health financing gains.

Countries of Eastern Europe and Central Asia have high rates of drug-resistant tuberculosis (TB), with nine countries in the region on the global list of 30 countries with highest burden of multidrug-/ rifampicin-resistant (MDR/RR) TB [1]. In contrast to global patterns, in the European region drug resistance is common among both new (24%) and previously treated (54%) cases, attributed to both suboptimal treatment and transmission of drug-resistant strains of *Mycobacterium tuberculosis* [5]. In the years of economic and political transition following the collapse of the Soviet Union, homelessness and poverty, accelerated drug trade and injecting drug use, high incarceration rates and prison overcrowding, and common labour migration to the Russian Federation became widespread and collectively exacerbated the spread of TB and drug-resistant TB in Eastern Europe and Central Asia [6,7]. Marginalization, variable access to healthcare, and poor living and working conditions have contributed to spread of TB among migrant workers [8]. Hospital-based health systems and related financing are a legacy of the former Soviet Union [9], leading to continued dependency on hospitalization for TB treatment in many countries [10,11]. Previous work has highlighted barriers to adopting ambulatory TB treatment where local health financing favours bed-based payment and lacks the mechanisms to transfer savings from hospitals to alternative care and support [12,13].

Broadly, Global TB strategy for TB prevention and care prioritize systems for early diagnosis of TB, treatment of all people with TB, preventive treatment for people living with HIV and household contacts of people with pulmonary TB, vaccination, and management of comorbidities [2,14–16]. WHO recommends preventive treatment for people living with HIV as well as household contacts. The latter includes children <5 years regardless of latent tuberculosis infection (LTBI)-status, and children >5 years and adults particularly when LTBI has been confirmed or treatment is otherwise indicated [16]. Treatment guidelines for drug-resistant TB have recommended ambulatory care since 2011 [15,17]. However, specific TB interventions, treatments and global guidance are rapidly evolving. Since 2022 global guidance has included the advent of WHO-recommended rapid diagnostics (WRDs) to improve the detection of TB and drug resistance using molecular technology and expanded options for shorter duration treatment for drug-resistant TB using novel all-oral 6-month regimens based on bedaquiline, pretomanid, linezolid and moxifloxacin (BpaLM) [15]. While many countries in Eastern Europe and Central Asia have begun using or piloting these innovations [18], not all recommended interventions are embedded in national policies [19].

There are persistent gaps in funding to support TB responses, and global funding for TB has decreased since 2019 [1]. Ideal allocative efficiency denotes the optimal distribution of resources between interventions, populations, and/or geographies to maximize population health outcomes, thus providing the best return on investment [20]. In countries with high burden of drug-resistant TB, such as Eastern Europe and Central Asia, allocative efficiency analyses can be highly valuable to inform the distribution of constrained resources to maximize progress towards End TB targets. Optima TB is an allocative efficiency model; that is, an epidemiological model capturing TB transmission between risk-disaggregated populations, intervention costs, and intervention impacts that is designed to optimise the distribution of funds between TB interventions to achieve the maximum impact on TB outcomes within a given budget level, subject to data on country-specific interventions. The optimised allocation will achieve the maximum impact through prioritising the most cost-effective combination of TB interventions. This analysis considers results from five independent Optima TB modelling studies conducted in 2023 in Eastern Europe and Central Asian settings to identify common and contrasting priorities in the region to reduce TB burden.

## Materials and methods

### Study overview and objectives

Individual modelling studies were undertaken between March and December 2023 in collaboration with country partners in Belarus, Kyrgyz Republic, Republic of Moldova, Tajikistan and Uzbekistan to inform national priority-setting and funding applications for TB [21–25]. The objectives of this cross-country analysis are:

1. To estimate the historical and future TB epidemic in five countries in Eastern Europe and Central Asia;
2. To identify common and contrasting priorities in TB spending across the region; and
3. To assess the projected benefits of optimised spending on projected TB epidemic outcomes relative to status quo.

### Contributing studies and country settings

Included modelling studies were set in countries ranging from approximately 3.2 million to 34.8 million in population size (median 9.6 million). Gross National Income (GNI) per capita ranged from $1210 to $7210 [26], with Belarus and Moldova classified as upper-middle income countries and Kyrgyz Republic, Tajikistan and Uzbekistan classified as lower-middle income countries [27].

The aim of individual studies was to assess the most cost-effective resource allocation across existing and prospective TB prevention, screening and treatment modalities to minimise TB burden by 2030. TB epidemic models were developed for each participating country using Optima TB (Atomica version 1.27.0). Optima TB is a dynamic, population-based compartmentalized model of TB transmission and disease progression integrated with an economic and programmatic analysis framework described in Goscé, Abou Jaoude (2021) and detailed in S1 Text. The epidemic model partitions the population by age and risk group, TB health state (i.e., susceptible, vaccinated, latent TB, active TB), diagnosis and drug resistance types (DS, MDR, XDR), and tracks people’s movement among health states [28]. Using user-defined relationships between cost, coverage, and outcome in combination with the epidemic model, Optima TB applies a mathematical optimization algorithm to determine how resources can be optimally allocated across different TB interventions to advance epidemic progress at specified funding levels.

### Data inputs for model parameterisation

Demographic, epidemiological, intervention and cost data were collated by the study team in collaboration with representatives from National TB Programmes. Key input parameters and sources are described in Table 1 and Table B in S2 Text. Model inputs were reviewed and validated by representatives from each country.

**Table 1:** Key input data parameters and common data sources.

Data type	Required parameters and key sources
Epidemiologic data	Demographic data for population size, birth rate estimates and all-cause mortality predominantly sourced from UN population division [30]; UNAIDS Spectrum estimates for people living with HIV population size [31]; prisoner population estimates from World Prison Brief [32] or country-provided data. TB notifications and TB-related deaths by population supplied by National TB Program for available years. Historical notifications from 2000 based on WHO-reported data without detailed disaggregation by population group [5]. Pulmonary TB only reported in Kyrgyz Republic and Moldova; other countries provided data on extrapulmonary and pulmonary TB.
Intervention coverage data	Treatment initiations and outcomes by sputum smear status (if available) and strain (DS, MDR, XDR), number of BCG vaccinations, and TB preventive treatment and latent TB infection treatment initiations (combined) supplied by National TB Programs for available years. Number of people screened by modality and positive yield informed by and National TB Program data and existing reviews of the National Tuberculosis Programmes, where available.
Cost data	Annual cost per treatment initiation provided by National TB Program, 2022, based on weighted cost of included treatment regimens by drug resistance status. Included costs generally incorporated treatment drugs, inpatient and outpatient care, on-treatment laboratory monitoring, adverse event management, psychosocial support, and other non-specified costs. Cost per person diagnosed or screened were derived from costs for TB diagnosis provided by National TB Program, 2022, number screened and estimated yield for active case finding modalities based on national data or alternative sources from published literature. Passive screening and prisoner screening services generally utilized cost per total population alive.

Notes: DS, drug susceptible; MDR, multi-drug resistant; TB, tuberculosis; UN, United Nations; WHO, World Health Organization; XDR, extensively drug-resistant

In all settings, baseline TB spending for 2022 was calculated from bottom-up estimates based on unit cost ×coverage for each included TB intervention. National TB Programmes provided unit cost estimates incorporating commodities, staff time, and intervention delivery costs, although actual inclusions may vary. Indirect and non-targeted spending (e.g., management, overheads, strategic information) as well as societal costs (e.g., lost productivity) were not included. Most countries reported spending in US dollars (US$), while Moldova reported costs in Euros. For comparability, all spending is reported in US$ in this multi-country analysis, with conversion from Euros based on 2022 average exchange rate [29].

Population risk groups were adapted to each country context and available data but generally included groups for the general population aged 0–4, 5–14, 15–64, and 65 + , people living with HIV on antiretroviral therapy, people living with HIV not on treatment (including those not yet diagnosed with HIV), prisoner populations, and male migrant workers. In Kyrgyz Republic people living with HIV were disaggregated by age group rather than treatment status.

Current and prospective interventions for TB prevention, diagnosis and treatment were defined in each country in collaboration with National TB Programmes based on relevance, national strategic priorities and data availability (Table A in S2 Text). For each intervention, total spending, unit cost, coverage, saturation and capacity constraints were defined (Table B in S2 Text). Saturation is the maximum achievable coverage as a proportion of the target population compartment that could be reached by an intervention in a given year. Intervention groups included in the multi-country comparisons are described in Table 2.

**Table 2:** Intervention groups included in multi-country comparisons.

	Description
**Prevention**	
BCG vaccination (BCG)	Bacillus Calmette-Guerin (BCG) vaccine to prevent the development of more serious forms of TB in children and often included in neonatal/infant vaccination schedule
TB preventive treatment for contacts (TPT contacts)	Treatment of asymptomatic household contacts or non-household repeat contacts of individuals with TB disease with a course of one or more anti-tuberculosis medicines with the intention to reduce progression to active TB
TB preventive treatment for people living with HIV (TPT PLHIV)	Treatment of people living with HIV who are unlikely to have TB disease with a course of one or more anti-tuberculosis medicines to reduce progression to active TB as part of a comprehensive package of HIV care.
**Diagnosis**	
Mass screening and mandatory testing	Systematic screening conducted among the general population regardless of TB risk, exposure or symptoms.
	Mandatory testing incorporates legally required testing of individuals such as healthcare workers, military conscripts, higher education students, and personnel working personnel working in pre-schools or public services.
Passive and other case finding	Symptomatic screening based on individuals presenting with symptoms at healthcare facilities and other case finding not elsewhere defined.
Contact tracing	Systematic screening among child and adult household contacts and other close contacts of individuals with TB disease. In Tajikistan, includes community contacts.
Active case finding in prisons (ACF prisoners)	Systematic screening for TB disease conducted in prisons and penitentiary institutions, such as at entry, annual screening, and screening upon release.
Active case finding among people at higher-risk, including people living with HIV (ACF people at higher risk, including PLHIV)	Targeted screening among populations at higher-risk of TB, which could include individuals with specific comorbidities (e.g., HIV, diabetes mellitus) and those with structural risk factors such as migrants, populations experiencing homelessness, and people who inject drugs. In Moldova, also includes people in prisons.
Community-based active case finding (ACF community/mobile screening)^*^	Targeted case finding in community-settings, including mobile screening units and through non-governmental organisations, intended to reach priority and harder-to-reach populations with high burden of TB, such as migrants, people experiencing homelessness and people with alcohol use disorders.
Active case finding through health facilities (ACF facility)	Systematic screening conducted in hospital and primary health care facilities to reach individuals with specific comorbidities (e.g., HIV, diabetes mellitus) or indications of TB disease (incorporates some passive case finding).
**Treatment**	
DS-TB treatment	Standard six-month treatment regimen
MDR-TB standard treatment	Standard treatment of 18–20 month duration
MDR-TB shorter treatment regimens	Includes modified shorter treatment regimens (mSTR, 9-month duration) and newer all-oral six-month regimen for drug-resistant TB using BPaLM (bedaquiline, pretomanid, linezolid and moxifloxacin) (5).
XDR-TB standard treatment	Standard treatment of 20 month duration.
XDR-TB shorter treatment regimens	Novel all-oral 6–9 month regimen composed of bedaquiline, pretomanid, linezolid (BpaL) or other treatment with shorter duration than standard regimens.

Notes: ACF, active case finding; BCG, Bacillus Calmette-Guerin; BpaL, bedaquiline, pretomanid, linezolid; BPaLM, bedaquiline, pretomanid, linezolid and moxifloxacin; DS, drug susceptible; MDR, multi-drug resistant; NGO, non-governmental organizations; PLHIV, people living with HIV; TB, tuberculosis; TPT; TB preventive treatment; XDR, extensively drug-resistant.

* Includes overlap with ACF among people at higher risk due to differing definitions of screening modalities between countries, and inclusion of separate modalities for community-screening and targeted screening among specific population groups in some settings.

Treatment for drug resistant TB (DR-TB) were classified by usual regimen length. Standard treatment regimens are usually for a duration of 18–20 months total. Shorter treatment regimens for DR-TB include six- and nine-month regimens.

### Model calibration

Each model was calibrated to available epidemiologic data on TB case notifications and WHO estimated TB incidence (Global TB Programme 2023 estimates) with the aim to align to estimates of key TB indicators such as active-TB incidence and prevalence, latent TB prevalence and TB-related deaths. Specific calibration parameters such as relative population susceptibility, were adjusted to closely match indicators including TB incidence and prevalence. Calibration methods and key calibration figures are presented in S3 Text. Cost-functions were defined for each intervention based on the relationship between spending, coverage and outcome.

### Scenarios considered

For each country, a baseline scenario projected outcomes over 2024–2030 with spending for each TB prevention, screening and treatment intervention maintained at 2022 levels.

Optimization scenarios were constructed to determine how spending could be reallocated between modelled TB interventions to minimise TB incidence and TB-related deaths. This was done for total amounts of funding equal to 75%, 100%, 125% and 150% of baseline TB spending. The objectives of the optimizations were to reduce, by equal proportions, the prevalence of DS-TB, MDR-TB and XDR-TB and the number of TB-related deaths across 2024–2030. This optimisation objective was selected instead of incidence to prioritise more rapid treatment of active TB and thus prevention of TB infection in the short term. In turn, this leads to longer-term reductions in active TB incidence. Weightings were specific to each country based on the estimated baseline conditions of 2022 (Table A in S3 Text). Each scenario assumes that changes in intervention coverage occur in 2024 and are sustained until 2030.

Changes in funding to achieve optimised allocations did not consider reallocation of care costs between hospitalized and ambulatory treatment modalities. Spending was constrained to not reduce current coverage of BCG vaccination and preventive therapy for people living with HIV, and to not reduce current spending on any program by more than 50%. These constraints were advised by the country teams based on equity considerations, spending funded outside of the TB program, and the risk of impacting non-TB related benefits associated with each intervention.

### Outcomes

This analysis collates modelling outputs on baseline spending patterns, optimised resource allocation and projected health benefits of 100% and 150% spending optimised relative to baseline spending from the five participating countries. Additional findings are available in country-specific reports (available at https://optimamodel.com/tb/applications.html) [21–25].

For baseline and optimised spending scenarios, annual and cumulative number of new/relapse pulmonary TB infections, pulmonary TB incidence per 100,000 population, and number of TB-related deaths were projected from 2024 to 2030 in each country. Number and proportion of incident pulmonary TB infections and TB-related deaths averted from 2024 to 2030, and number of additional people that could be treated, were estimated relative to the baseline spending scenario. Best estimates for cumulative outcomes are based on the modelled impact of changed spending for calibrated parameter sets. The uncertainty range is based on the 95% confidence interval from pairwise differences between parameter sets sampled with the same random seed, given changes in spending.

### Ethics statement

This work does not report on studies involving humans and related data was not subject to ethical review.

## Results

### TB epidemiological situation

**Fig 1:**
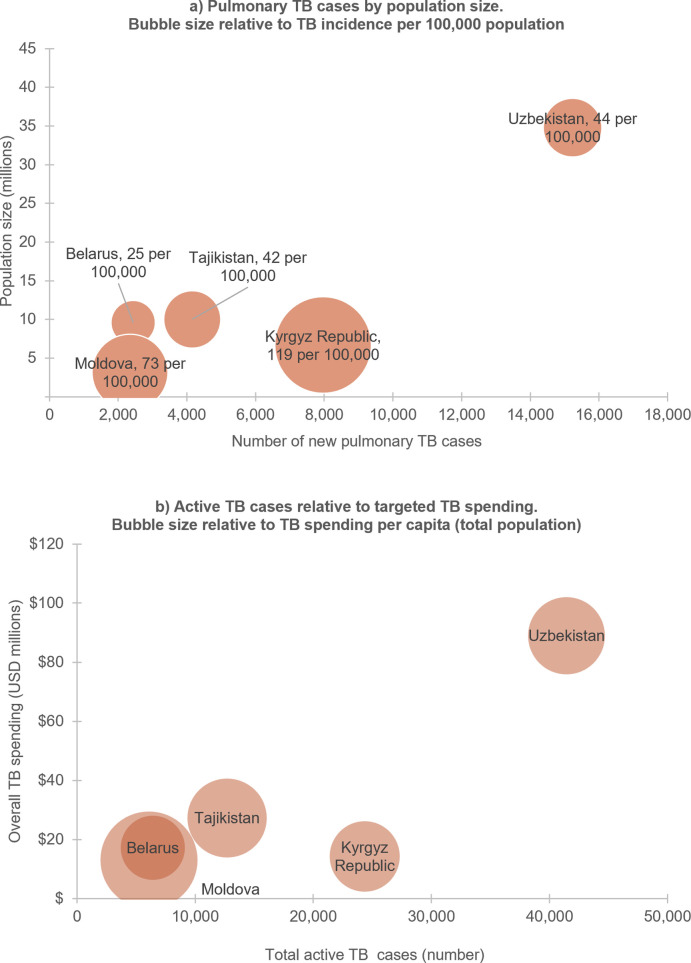
TB burden and spending by country. Panels show: **a)** Pulmonary TB cases in relation to population size in participating countries, 2022; and **b)** TB spending relative to size of TB epidemic measured through total active TB cases. Sources: Optima TB 2023 country-specific models.

Optima estimated pulmonary TB incidence ranged from 25 per 100,000 population in Belarus to 119 per 100,000 in Kyrgyz Republic (Table 3). In absolute terms, Uzbekistan had the highest number of estimated new and relapse pulmonary TB cases in 2022, at 24,900, in line with higher overall population size (Fig 1). Where both pulmonary and extrapulmonary TB were modelled, pulmonary TB accounted for 52% of incident cases in Tajikistan, 61% in Uzbekistan, and 87% in Belarus. In all five countries, prisoner populations were disproportionately affected by TB, with an average estimated pulmonary TB incidence of 1151 per 100,000 population. Where modelled, people living with HIV and migrant workers also had high incidence of pulmonary TB, with an average incidence rate of 885 and 281 per 100,000 population, respectively, compared to 46 per 100,000 population among the general adult population in all countries. Estimated new and relapse TB cases with drug resistance ranged from 14% in Tajikistan to 43% in Belarus. TB-death rate per 100,000 populations was highest in Tajikistan, at 9.6 per 100,000, and lowest in Belarus, at 3.7 per 100,000.

**Table 3:** Optima TB model estimates of TB epidemiological burden in participating countries, 2022.

	Belarus	Kyrgyz Republic^a^	Moldova^a^	Tajikistan	Uzbekistan
**Country description:**					
Population size	9,600,000	6,700,000	3,200,000	10,000,000	34,800,000
GNI per capita (current US$)	7,210	1,440	5,500	1,210	2,190
**HIV burden:**					
HIV adult prevalence	0.4%	0.3%	0.9%	0.2%	0.2%
Estimated number of people living with HIV	27,000	11,000	16,000	15,000	82,000
**Modelled TB burden:**					
Total TB incidence, number (IQR)	2,795	N/A^a^	N/A^a^	8,026	24,939
	(2,285-3,287)	0	0	(6,580-9,836)	(18,531-35,470)
Pulmonary TB incidence, number (IQR)	2,432	7,966	2,347	4,153	15,231
	(2,010-2,850)	(6,954-9,463)	(1,906-2,956)	(3,370-5,132)	(11,795-21,186)
Pulmonary TB incidence, rate per 100,000 population (IQR)	25	119	73	42	44
	(21-30)	(104-142)	(61-94)	(34-51)	(34-61)
DR-TB incidence, number (IQR)	1,211	2,461	492	1,150	3,837
	(1,029-1,434)	(2,191-2,961)	(412-625)	(932-1,433)	(2,854-5,544)
% of DR-TB among incident TB cases (IQR)	43%	31%	21%	14%	15%
	(31%-63%)	(23%-43%)	(14%-33%)	(9%-22%)	(8%-30%)
Total active TB cases, number (IQR)	6,410	24,351	6,091	12,700	41,446
	(4,983-7,947)	(20,443-28,347)	(4,595-8,505)	(9,964-15,864)	(28,258-61,515)
Latent TB prevalence (IQR)	14%	12%	17%	11%	18%
	(14%-15%)	(11%-13%)	(16%-19%)	(11%-12%)	(18%-19%)
TB-related deaths, number (IQR)	355	610	285	958	2,666
	(286-443)	(441-773)	(258-425)	(703-1,259)	(1,775-4,299)
TB-related deaths, rate per 100,000 population (IQR)	3.7	9.1	8.9	9.6	7.7
	(3.0-4.6)	(6.6-11.6)	(8.2-13.5)	(7.0-12.6)	(5.1-12.4)
Pulmonary TB incidence by population group, rate per 100,000 population (IQR):					
Prisoners	1,621	1,830	2,038	549	356
	(1,457-1,858)	(1,653-1,954)	(1,537-3,002)	(203-808)	(279-814)
Migrant workers	N/A	N/A	256 ^b^	137	N/A
			(242-283)	(110-160)	
People living with HIV	182	N/A	872	994	1,132
	(125-233)		(665-1,088)	(805-1,156)	(1,092-1,287)
Other modelled populations^c^	19	118	60	35	41
	(15-24)	(102-141)	(48-81)	(27-44)	(31-59)

Notes:

^a^Modelled pulmonary TB only;

^b^Number highly influenced by changes in migration patterns due to the war in Ukraine and may not be representative;

^c^ Based on children and adults not defined as people living with HIV, prisoners or migrants; DR, drug resistant. Interquartile range (IQR) used to represent uncertainty intervals as these most closely reflected WHO uncertainty intervals for data used in calibration.

Sources: Optima TB 2023 country-specific models

### Baseline TB spending

Across the five countries, average estimated TB spending per capita was $2.66. With the exception of Kyrgyz Republic, overall targeted TB spending generally corresponded to the size of the epidemic, with highest overall spending in Uzbekistan, at $89 million (Fig 1). Spending in Kyrgyz Republic was lower relative to the size of its TB epidemic, reflecting a combination of higher TB incidence rates per capita, lower GNI, and a significant loss of resources for TB due to COVID-19-related disruptions as of the 2022 baseline year.

**Fig 2:**
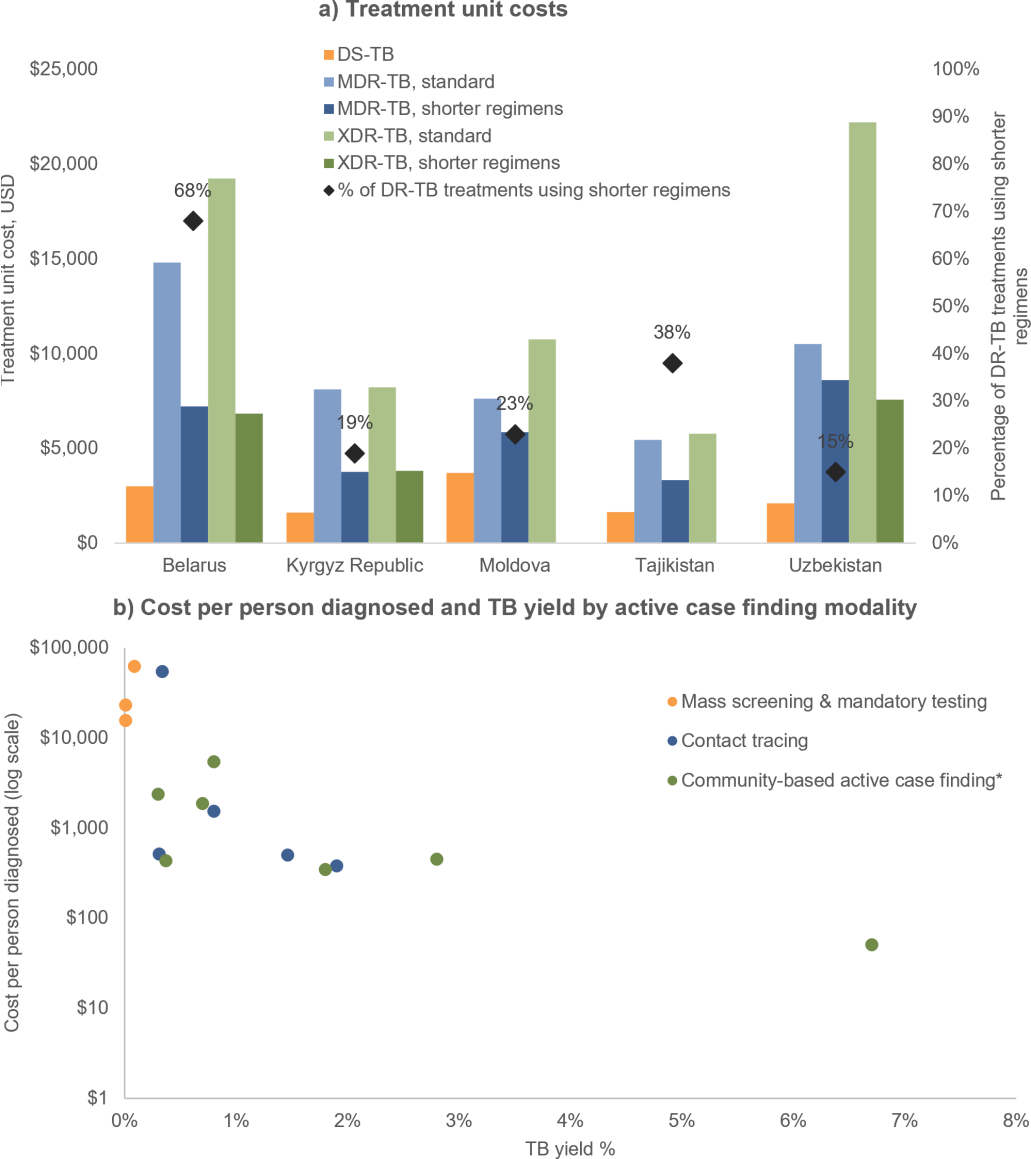
Treatment and screening unit costs. Panels show: **a)** Treatment unit cost by country, TB strain and regimen duration; and **b)** Cost per person diagnosed (log scale) and TB yield by active case finding modality group. Notes: * Includes active case finding through community-based organisations, targeted mobile screening, and among populations at high risk of TB. Screening modalities are grouped by type, and more than one modality may be plotted for a given country. For example, three forms of community-based active case finding (ACF) were modelled in Moldova: ACF through community-based organisations, targeted mobile screening, and ACF among populations at high risk of TB. Mass screening/mandatory testing not modelled in all countries. Costs in US dollars. DR, drug resistant; DS, drug susceptible; MDR, multi-drug resistant; TB, tuberculosis; XDR, extensively drug-resistant. Source: Optima TB country-specific models, 2023.

Treatment accounted for the majority of TB spending (63%-87%) with the exception of in Tajikistan (32%), which had a very large contact tracing program that accounted for 57% of spending. Shorter treatment regimens for MDR-TB and XDR-TB had lower unit costs than standard treatment regimens (Fig 2A). Use of shorter treatment regimens for DR-TB was highest in Belarus (68%) and lowest in Uzbekistan (15%). Accounting for the reported use of standard and shorter treatment regimens, the weighted average treatment costs across participating countries were $2,407 for DS-TB, $7,651 for MDR-TB and $11,856 for XDR-TB (Table 4).

**Table 4:** Overall TB spending (US$) and spending by selected TB interventions by country, 2022.

	Belarus	Kyrgyz Republic	Moldova	Tajikistan	Uzbekistan
**Overall TB spending**	$17,199,025	$14,250,383	$13,089,317^a^	$27,252,434	$88,957,003
**Mass screening**	$5,536,503	$0	$0	$407,202^b^	$10,919,288^c^
** % of overall spending**	32%	0%	0%	1%	12%
** % TB yield**	0.006%	NA	NA	0.07% (school-based), 0.1% (TB days)	0.01%
** Cost per person diagnosed**	$23,185	NA	NA	$62,647	$15,475
**Contact tracing**	$10,954	$136,865	$187,047	$15,398,122^d^	$461,082
** % of overall spending**	0%	1%	1%	57%	1%
** % TB yield**	0.31%	1.46%	0.8%	0.08% (adults), 0.59% (children)	1.9%
** Cost per person diagnosed**	$513	$505	$1,533	$54,812	$380
**Active case finding in community and among people at higher risk** ^e^	$3,761	$13,955	$3,214,488^f^	$217,837	$4,265,659
** % of overall spending**	0.0%	0.1%	25%	1%	5%
** % TB yield**	0.37% (PWID)	6.7% (PLHIV)	2.8% (mobile), 0.8% (NGO), 0.7% (high risk)	1.8% (NGO)	0.3% (people at high risk)
** Cost per person diagnosed**	$436	$51	$454 (mobile), $5437 (NGO), $1874 (high risk)	$349	$2,363
**TB preventive treatment**	$14,066	$60,332	$10,489	$1,133,870	$640,785
** % of overall spending**	0.1%	0.4%	0.1%	4%	1%
** Cost per person treated** ^g^	$11.52	$31.49-$117.41	$3.16-$7.10	$3.36-$406	$3.36-$32.52
**TB treatment**	$11,352,094	$12,460,624	$9,262,557	$8,740,105	$56,354,596
** % of overall spending**	66%	87%	71%	32%	63%
** Cost per person treated, DS-TB**	$2,993	$1,598	$3,718	$1,627	$2,101
** Cost per person treated, MDR-TB**	$8,705	$7,258	$7,420	$4,625	$10,247
** Cost per person treated, XDR-TB**	$15,576	$8,212	$10,769	$5,767	$18,956

Notes: DR, drug resistant; DS, drug susceptible; MDR, multi-drug resistant; NA, not available; TB, tuberculosis; XDR, extensively drug-resistant. All spending reported in US dollars (US$).

^a^Spending provided in euros and converted to US$ based on average exchange rate in 2022 (1€: $1.05).

^b^Includes mass screening through schools and community TB days. Modalities disaggregated in model.

^c^Based on mandatory testing.

^d^Disaggregated by contact tracing among child and adult contacts in model.

^e^Includes screening through non-governmental organisations (NGOs), targeted mobile testing and screening among populations at higher risk of TB.

^f^Modelled as three separate modalities for targeted mobile testing, ACF through NGOs, and ACF among other populations at risk, and includes some testing in prisons.

^g^Range in cost for treating different subgroups (i.e., household contacts by age group and people living with HIV) presented.

Source: Optima TB country-specific models, 2023

Cost per person diagnosed varied by country and modality but were lower for community-based active case finding and contact tracing than mass screening in all countries (Fig 2B). The highest TB yields were reported for screening among people living with HIV in Kyrgyz Republic (6.7%) and targeted mobile screening in Moldova (2.8%), while mass screening and mandatory testing had the lowest TB yields. The cost per person diagnosed through contact tracing ranged from $380 in Uzbekistan to $1533 in Moldova, with an outlier of $54,812 in Tajikistan where contact tracing included community screening for active TB contacts outside of the household. The cost per person diagnosed through mass screening ranged from $1,340 in Uzbekistan (category also included passive case finding) to $62,647 in Tajikistan.

### Optimised spending allocation

**Fig 3:**
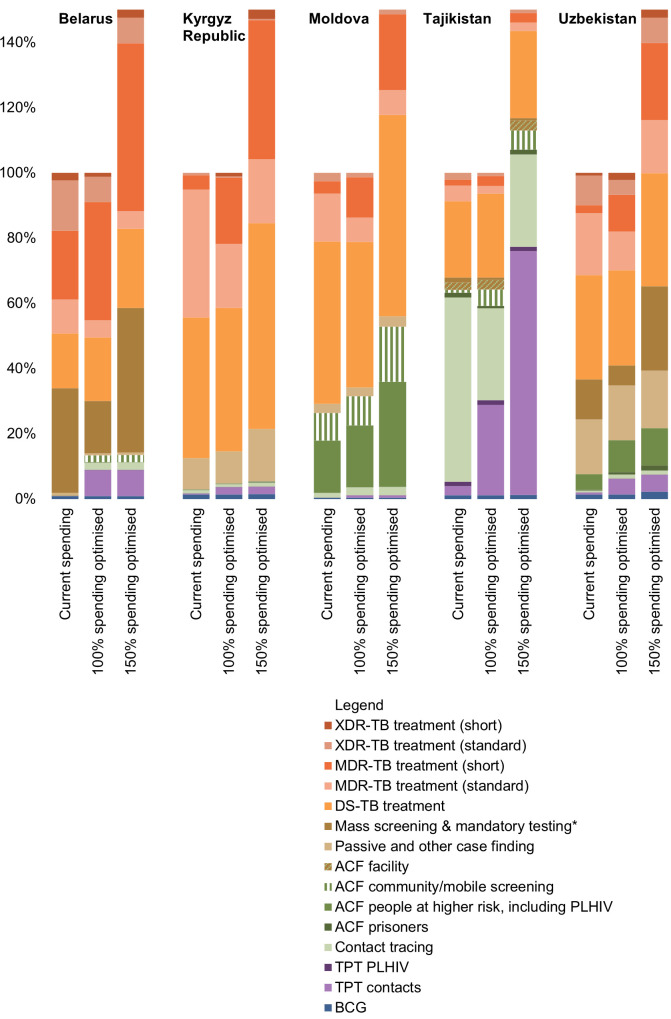
Allocation of funding by TB intervention relative to total TB spending in baseline spending, 100% and 150% spending optimised to minimize drug-resistant TB and TB-related deaths. Notes: ACF, active case finding; BCG, Bacillus Calmette-Guérin; DS, drug susceptible; MDR, multi-drug resistant; PLHIV, people living with HIV; TB, tuberculosis; TPT, TB preventive treatment; XDR, extensively drug-resistant. * Mandatory testing considered in Uzbekistan only. Source: 2023 Optima TB country models.

**Fig 4:**
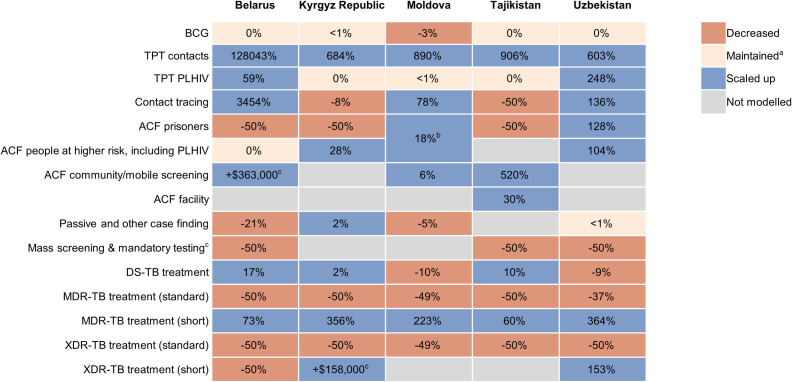
Percentage change in spending allocation with 100% spending optimised relative to baseline spending by TB intervention for five participating countries. Notes: a, Baseline spending maintained or less than 1% increase in baseline spending; b, prisoners included in intervention for ACF among populations at higher risk; c, No spending in baseline so absolute value of spending allocation shown; c, Mandatory testing only modelled in Uzbekistan. ACF, active case finding; BCG, Bacillus Calmette-Guérin; DS, drug susceptible; MDR, multi-drug resistant; PLHIV, people living with HIV; TB, tuberculosis; TPT, TB preventive treatment; XDR, extensively drug-resistant. Source: 2023 Optima TB country models.

In all countries, 100% spending optimised prioritized expanding shorter treatment regimens for DR-TB in place of standard regimens (Figs 3 and Fig 4), with standard treatment regimens reduced to the minimum constrained 50% of most recent reported spending reflecting challenges of rapid transition. The maintenance or reduction of treatment spending for DS-TB and XDR-TB in some settings was due to reducing incidence of these strains and the suitability of MDR-TB treatment regimens for most DR-TB given changing definitions of XDR-TB. At higher budget levels, shorter treatment regimens for DR-TB are further prioritized.

In terms of TB diagnosis, optimised 100% spending commonly reduced allocations for mass screening and mandatory testing to prioritize more targeted active case finding. Where modelled, this included scaling up community-based and mobile screening as well as focusing on populations at higher risk, including people living with HIV. Optimised spending prioritized expanding contact tracing in three of five countries. In two countries it was recommended to make contact tracing more cost-effective by focusing on select contacts. In Tajikistan this reflected the current, wider approach to community screening for contacts that could be made more cost-effective by first focusing on child household contacts for screening and TPT (Fig 4). Concurrently, their wider approach to community screening also facilitated the potential for TPT for adult contacts to be expanded at higher budget levels. In Kyrgyz Republic this reflected an opportunity to focus on TPT for identified child contacts through household tracing ahead of additional contact tracing. With budget level increased to 150% of baseline spending, mass screening and mandatory testing were scaled-up as more cost-effective case finding modalities were close to maximum achievable coverage with 100% spending optimised (Fig 3). Due to declining prison population size and/or TB incidence among prisoners, prison-based TB services were only prioritized for scale up in Uzbekistan and Moldova.

Passive and other case finding was heterogeneously defined between countries, but in most countries there was minimal scope to further optimise spending. BCG vaccination programs were maintained with high coverage in all countries, but spending decreased in Moldova due to declining population in need. Detailed spending allocations in baseline and optimised spending scenarios are provided in Table A in S4 Text.

### Impact of optimised spending

**Fig 5:**
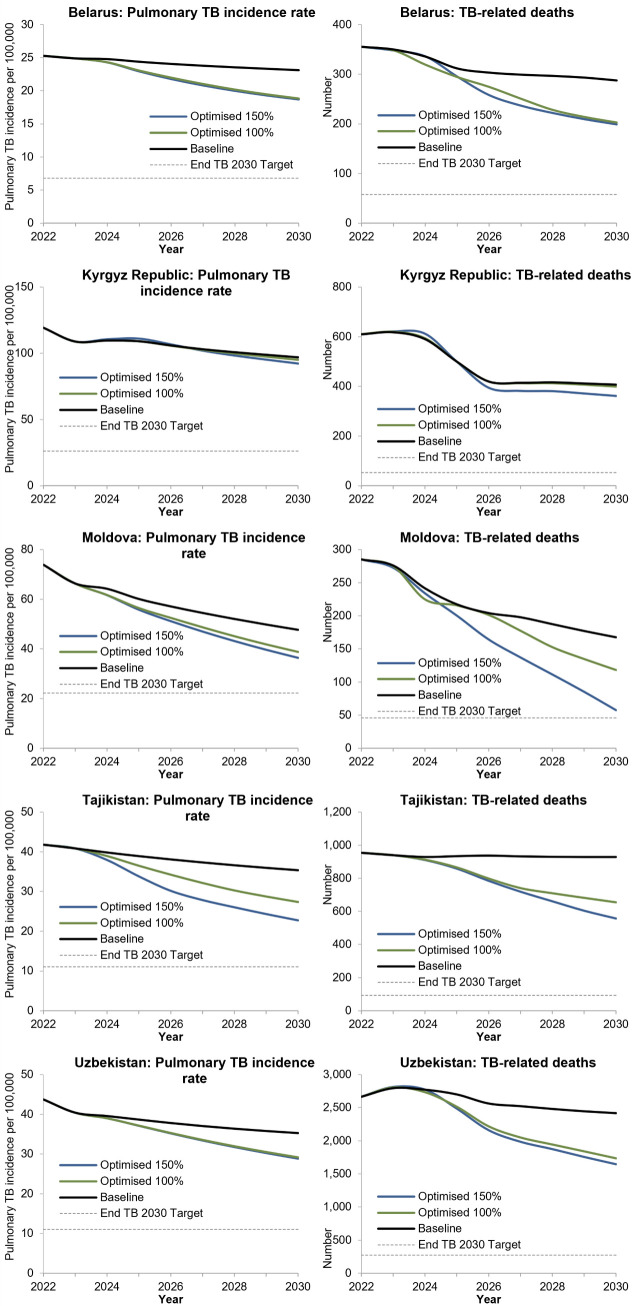
Projected change in pulmonary TB incidence and TB-related deaths from 2022 to 2030 with baseline spending continued, optimised 100% spending, and optimised 150% spending by country.

Excluding Kyrgyz Republic, it was estimated that 100% spending optimised could reduce the cumulative number of new/relapse TB infections by 9 − 13% and the cumulative number of TB-related deaths by 12 − 18% from 2024 to 2030 compared with if baseline spending were continued (Table B in S4 Text). By 2030, pulmonary TB incidence is projected to decrease to between 19 and 95 per 100,000 population in included countries with 100% spending optimised (Fig 5). No countries were projected to reach their End TB targets. Incidence reductions were possible through the expansion of TPT and reduced transmission on account of earlier diagnosis through targeted screening. Savings made by shifting from standard to shorter treatment regimens make it possible to treat more people for DR-TB and thus avert more deaths. The cumulative number of people initiating treatment for DR-TB could increase by 9 − 34% in modelled countries equating to an additional 200–2,400 people.

In Kyrgyz Republic, which had lower TB spending relative to size of epidemic (Fig 1), the projected impact of optimised spending is limited because following a COVID-19 related reduction in TB spending since 2019, the 2022 baseline represents primarily the maintenance of treatment for people diagnosed through passive case finding and other TB testing, with limited opportunities to reallocate additional spending.

In Tajikistan, the prioritization of further expansion of TPT among adult contacts with 150% spending optimised could avert 23% of new/relapse pulmonary TB infections compared to baseline spending (Fig 5). In Moldova it may be possible to reach End TB targets for reduction in TB-related deaths by 2030 with 150% spending with increased investment in shorter-treatment regimens for MDR-TB and expanded case finding. In Belarus and Uzbekistan, 150% spending optimised had little to no additional impact on TB incidence and TB-related deaths, with the primary difference being expansion of mass screening and mandatory testing at higher budgets.

## Discussion

### Summary of findings

Countries in Eastern Europe and Central Asia have some of the highest burdens of drug-resistant TB globally, with both health and financial implications. Of the five countries with unique epidemic profiles considered, none are projected to reach End TB targets with currently implemented interventions. There are opportunities to reallocate existing TB funds for higher epidemic impact by adopting shorter treatment regimens for DR-TB, prioritizing targeted screening among populations at higher risk, including household contacts of persons with active TB, and expanding TB preventive treatment. With these changes, across five countries it may be possible to avert a total of 4,600 deaths and 16,000 TB infections over 2024–2030 without additional resources. However, End TB targets may remain out of reach without new and prospective interventions.

### Findings in context

By optimizing resource allocation across current TB interventions, incremental reductions in TB incidence and mortality may be possible in Eastern Europe and Central Asia. However, many of these interventions are already close to maximum achievable coverage, thus limiting how widely they can be scaled up. While this study only considered changes in allocation of resources to existing TB interventions, there may be further opportunities for epidemic progress through technical efficiencies in program implementation such as use of artificial intelligence to support radiography interpretation through computer-aided detection (CAD) software [33], strengthening referral and laboratory linkages, enhanced integration of TB services with existing community-based services and relevant non-governmental organisations (NGOs), and embedded quality improvement processes may enable additional progress toward End TB targets [34]. Some of these may already be incorporated into current on prospective National TB Plans, thus this analysis may have underestimated reductions in TB incidence and deaths.

### Implications for treatment regimens

By the end of 2022, adoption of shorter treatment regimens varied across countries studied, with highest coverage in Belarus and lowest in Uzbekistan. The smaller TB epidemic and higher GNI per capita in Belarus may have facilitated greater uptake, as both countries participated in operational research studies for 6-month treatment regimens [35]. This modelling analysis showed that shifting to shorter treatment regimens is more cost-effective and can enable substantial increases in the number of people being treated for the same or less overall spending. Ensuring that the newest regimens are supported in national guidelines, expanding access to drug sensitivity testing, providing health worker training, and community demand generation could collectively facilitate implementation of newer regimens [36]. As seen with the difficulty of shifting from hospital-based to ambulatory TB treatment due to TB financing mechanisms, implications for hospital funding being reduced with adoption of shorter duration therapy may also pose a barrier to scaling up shorter treatment regimens in countries in Eastern Europe and Central Asia [12,13].

### Implications for TB preventive treatment

This analysis found that scaling up TPT for household contacts can have a substantial impact on reducing population-level TB incidence and was recommended in most settings. Underutilisation of TPT has previously been highlighted by global partners [37,38]. The public health benefits of TPT need to be weighed against individual risks of drug-related adverse events, probability of progression to active TB, and implementation costs [16]. Providing preventive treatment is much cheaper than treating active TB, thus the optimisation prioritised scaling up TPT among populations most at risk of developing TB disease. Although prevalence of LTBI is higher in adolescents >15 years and adults, children <5 years are at greater risk of progressing to active TB and of developing more severe outcomes [16], and TPT was more cost-effective for children than adults in all countries. However, with the exception of Tajikistan, TPT expansion for both child and adult contacts was prioritized within the current budget envelope in these modelling analyses. Broader expansion of TPT among household contacts may be more cost-effective and justifiable in these settings given the high cost and difficulty to treat drug-resistant TB [16]. In Tajikistan, TPT for child contacts is still prioritized, but the broader definition of contact tracing means that TPT for adult community contacts may be lower priority, given relatively lower prevalence of latent TB and drug-resistant TB. Although this analysis did not differentiate between different TPT regimens, prioritising newer rifamycin-based regimens (e.g., 3HP and 3TP) may facilitate the scale-up of TPT by improving acceptability, adherence and completion rates given shorter duration and reduced toxicity [39,40]. Healthcare provider training, community engagement, procurement systems to ensure sustainable supplies of diagnostic tests, and patient counselling may also help to overcome common barriers to TPT [41].

### Implications for TB screening

Case finding can be improved in all countries by focusing on screening strategies among populations at higher risk. Contact tracing is prioritized for scale-up in most settings. In some countries expanding contact tracing beyond household contacts to include repeat contacts from community settings could improve case finding in line with country targets, even when accounting for lower transmission rates outside of household contacts [42]. However, in Tajikistan contact tracing already included a high proportion of community contacts and was associated with low TB yield, and subsequently high cost per diagnosis. In this case, the model optimization projected higher epidemic impact by first focusing on screening child household contacts.

Targeted TB screening services delivered through NGOs and mobile screening units among communities with high burden of TB were more cost-effective than mass screening in all settings modelled. These modalities have potential to better reach priority groups−such as migrant workers, people experiencing homelessness and people who inject drugs−through community-based organisations with established relationships with these communities and ability to tailor strategies and overcome barriers to TB screening, such as stigmatization [43]. These differ to mass screening strategies that are made available in the community but are not focused on populations most-at-risk. Given relatively low TB yield, estimated cost per diagnosis through mass screening was 40–50 times higher than costs through contact tracing and targeted active case finding in Belarus; a similar difference was found between mandatory testing and contact tracing in Uzbekistan. Subsequently, mass screening services were only prioritized for expansion at higher budget levels, once more targeted screening modalities have been saturated. Elsewhere, evidence of cost-effectiveness of TB screening is strongest among people living with HIV, but screening may also be more cost-effective when focused on other groups with high burden of undiagnosed TB when utilising symptom screening or chest X-ray as first screen, followed by Xpert diagnosis [44]. Among the countries studied, there was evidence of high TB burden among people living with HIV and migrant workers, but there was insufficient data to define population size or burden of TB in other communities considered to be at higher risk. Further strategic information and detailed epidemiological data could facilitate more effective implementation of active case finding [45].

### Limitations

This analysis is subject to several limitations. These findings are based on five countries that were self-selected based on requests for support in budget optimization and may not be representative of the Eastern Europe and Central Asia region. Unit costs for some interventions were informed by limited data, and cost and spending estimates are subject to some levels of uncertainty. In particular, there were no recent data to inform TB-related unit costs in Kyrgyz Republic after 2018, and this may have contributed to lower spending relative to size of epidemic. Due to limited data available, diagnosis costs did not include full implementation costs, such as health staff and transport costs for mobile screening. These may impact relative cost-effectiveness between modalities. Unit costs per person diagnosed also do not take into account net health benefits and health opportunity costs, such as due to earlier diagnosis. Modelled population, interventions, costs and objectives were developed specific to each country, and some data may not be directly comparable. For instance, passive and other case finding was inconsistently defined according to data available in each country. Restricted access to prison populations resulting in high uncertainty in prison population size, TB screening coverage, and TB epidemiology in prisons may have impacted results. Given exceptionally high TB burden in the region, active case finding through prison settings is likely to remain relevant in these countries, but additional data may help to inform the most cost-effective approach to TB screening in prisons [44,46]. These analyses did not assess potential savings from shifting from hospital-based to ambulatory treatment for TB, though this has been the subject of previous Optima TB analyses [12]. Resource needs for treatment of drug resistant strains were projected based on the proportion of incident drug resistant cases in 2022, but this may continue to evolve based on either suitability of new drugs to treat previously XDR-TB cases as per the WHO reclassification of XDR in 2021 [47], or further emergence of new drug resistance.

## Conclusion

With current interventions for TB prevention, diagnosis and treatment in Eastern Europe and Central Asia, it may be possible to reduce TB infections and deaths through evidence-based resource allocation. Shifting from standard to shorter treatment regimens, enhancing case-finding through contact tracing and targeted community-based testing strategies, and scaling up preventive treatment are likely to be the most impactful in this region. However, the End TB targets are likely to remain out of reach unless new and prospective interventions are implemented.

## Supporting information

S1 Text Model description. (DOCX)

S2 Text Data inputs and parameters. (DOCX)

S3 Text Calibration methods and figures. (DOCX)

S4 Text Detailed findings by country. (DOCX)
