# Supplementary material for: Optimising TB investments in Belarus, Moldova, Kyrgyz Republic, Tajikistan and Uzbekistan: An allocative efficiency analysis
Source: PLOS Glob Public Health. 2025 Jul 11;5(7):e0004548. doi: 10.1371/journal.pgph.0004548 (PMC12250568; doi:10.1371/journal.pgph.0004548)
Supplement: S1 Text — (DOCX) [file pgph.0004548.s001.docx]

# S1. Model description

The Optima TB tool is based on a dynamic, population-based TB model encapsulated within an intervention and costing framework [1]. The model uses a linked system of ordinary differential equations to track the movement of people among health states (Fig A). The overall population is partitioned in two ways: by population group and by TB health state. TB infections occur through the interactions among different populations. Each compartment in Fig A corresponds to a single differential equation in the model, and each rate (Fig A arrows) corresponds to a single term in that equation. The model then must be calibrated so that the model generates accurate estimates of notified TB cases, TB incidence, TB prevalence, the number of people on treatment, and any other epidemiological data that are available (such as TB-related deaths). Model calibration and validation normally should be performed in consultation with governments in the countries, in which the model is being applied.

Fig A.Optima TB model diagram


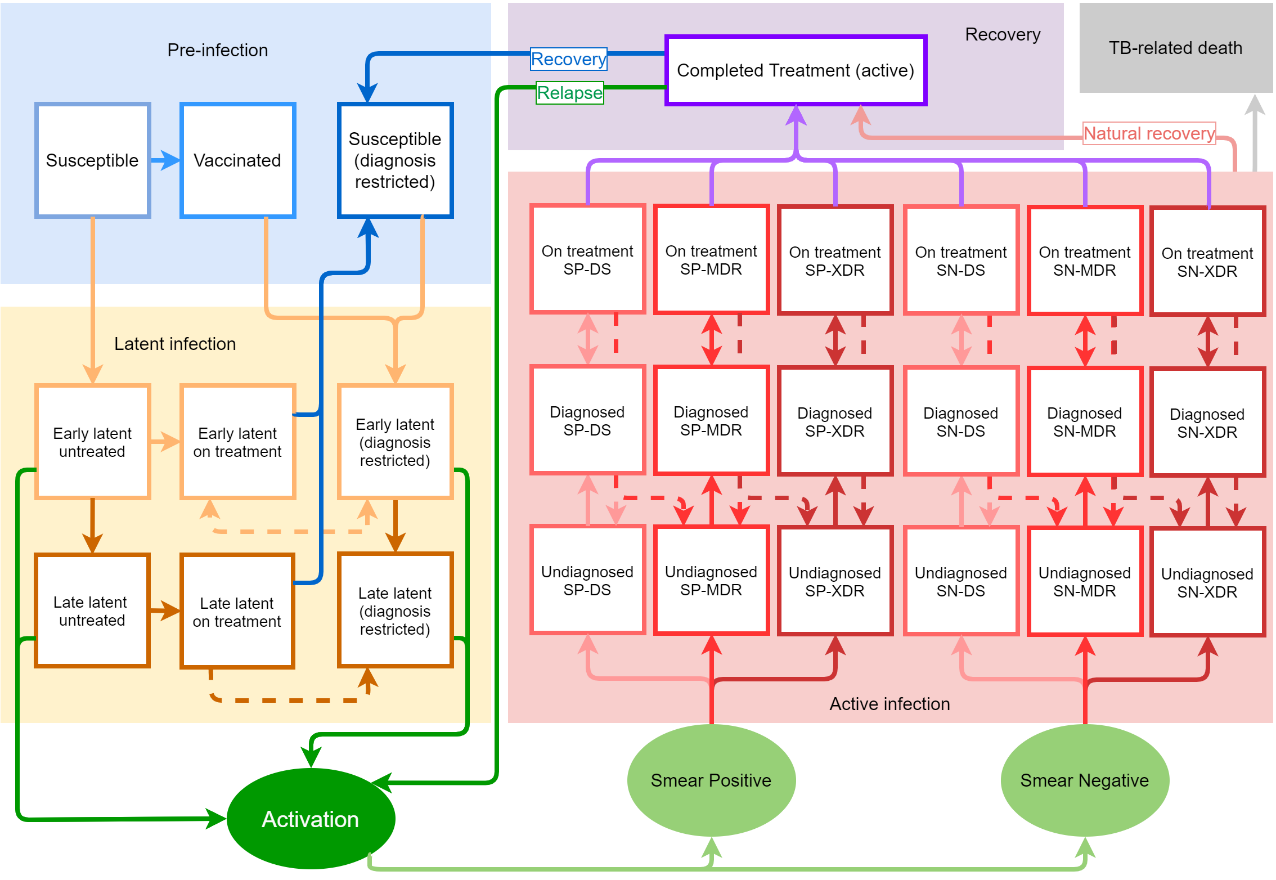
Source: Goscé, Abou Jaoude (2021)

Note: Each compartment represents a single population group with the specified health state. Each arrow represents the

movement of numbers of individuals between health states. All compartments except for “susceptible” and “vaccinated”

represent individuals with either latent or active TB. Death can occur for any compartment, but TB related mortality varies

between compartments. SN-DS, smear-negative drug susceptible; SP-DS, Smear-positive drug susceptible;

SP-MDR =smear-positive multi-drug resistant; SN-MDR, smear-negative multi-drug resistant; SN-XDR, smear-negative

extensively drug-resistant; TB, tuberculosis.

The WHO definition for incident TB cases includes both new and relapse cases. In the model, incident TB cases correspond to the following transitions between compartments:

- New cases: these are represented by the number of progressions to active TB from early and late latent-TB compartments. ‘New’ also includes recurring episodes of TB from the recovered compartment following re-infection
- Relapse cases: these correspond to a new episode of TB disease after previous completion of treatment or natural recovery.

Treatment success includes ‘cured’ and ‘treatment completion’, as per the WHO definition:

- Death during TB treatment is not included in treatment failure, but is considered separately
- Treatment failure and ‘loss to follow-up’ during treatment are included as separate outcomes in the model.
