# Supplementary material for: Optimising TB investments in Belarus, Moldova, Kyrgyz Republic, Tajikistan and Uzbekistan: An allocative efficiency analysis
Source: PLOS Glob Public Health. 2025 Jul 11;5(7):e0004548. doi: 10.1371/journal.pgph.0004548 (PMC12250568; doi:10.1371/journal.pgph.0004548)
Supplement: S2 Text — (DOCX) [file pgph.0004548.s002.docx]

# S2. Data inputs and parameters

Data inputs and parameters used in each analysis are available in country-specific reports and are summarized in the tables below [1-5].

Table A. TB prevention, diagnosis and treatment modalities implemented in each country model

|  | **Belarus** | **Kyrgyz Republic** | **Moldova** | **Tajikistan** | **Uzbekistan** |
| --- | --- | --- | --- | --- | --- |
| **Prevention** |  |  |  |  |  |
| BCG vaccination | **X** | **X** | **X** | **X** | **X** |
| TB preventive treatment (TPT) for contacts | **X**  0-4,5-14,15-64 | **X**  0-4, 5-14 | **X**  0-4,5-14,15-17,18-64, 65+ | **X**  0-4, 5-14, 15-64 | **X**  0-4, 5-14, 15+ |
| TPT for PLHIV | **X** | **X** | **X** | **X** | **X** |
| TPT for non-household repeated contacts all ages^1^ |  |  |  |  | **X^1^** |
| **Diagnosis** |  |  |  |  |  |
| Household contact tracing | **X** | **X** | **X** | **X** | **X** |
| Active case finding among prisoner populations | **X** | **X** |  | **X** | **X** |
| Active case finding (populations at high-risk) |  | **X**  PLHIV | **X**  Includes prisoners, migrants and PLHIV |  | **X** |
| Active case finding (community-based) | **X** |  | **X**  NGO | **X**  Includes mobile outreach and screening delivered through NGOs |  |
| Active case finding (mobile outreach) |  |  | **X** |  |  |
| Active case finding (mandatory testing) |  |  |  |  | **X** |
| Active case finding (hospital and primary health care) |  |  |  | **X** |  |
| Mass screening | **X** |  |  | **X**  School-based; Community TB day | **X**  Other case finding including mass testing and passive case finding |
| Passive case finding | **X**  Includes passive case finding | **X**  Other case finding not defined including passive case finding | **X** | **X** |  |
| Other testing |  |  |  |  |  |
| **Treatment** |  |  |  |  |  |
| DS-TB treatment | **X** | **X** | **X** | **X** | **X** |
| MDR-TB standard treatment | **X** | **X** | **X** | **X** | **X** |
| MDR-TB shorter treatment regimens | **X** | **X** | **X** | **X** | **X** |
| XDR-TB standard treatment | **X** | **X** | **X** | **X** | **X** |
| XDR-TB shorter treatment regimens | **X** | **X^1^** |  |  | **X** |

Notes: 1, Prospective intervention; BCG, Bacillus Calmette-Guerin; DS, drug susceptible; MDR, multi-drug resistant; NGO, non-governmental organizations; PLHIV, people living with HIV; TB, tuberculosis; TPT; TB preventive treatment; XDR, extensively drug-resistant

Table B. Modelled capacity constraints

| **BELARUS** | | | | | |
| --- | --- | --- | --- | --- | --- |
| **TB program** | **Coverage definition used** | **Baseline coverage 2022** | **Minimum constraint *** | **Maximum constraint** | **Notes** |
| BCG vaccination | Number vaccinated | 106,813 | 86,052 |  | Minimum constraint based on maintenance of BCG vaccination for estimated total births through maternal, newborn, and child health programs. |
| TB preventative therapy for 0-4 | Number treated | 0 |  | 1,004 | Constraint on maximum expansion of contact tracing based on: expected number of index cases, allowing for an increase in case-finding; average number of contacts tested per index case (2.8); number of contacts to be tested per index case to meet national targets for contact tracing among child contacts under 5 (6.2); and modelled TB prevalence among household contacts by age group. Sources: [6,7] |
| TB preventative therapy for 5-14 | Number treated | 15 |  | 2,511 |  |
| TB preventative therapy for 15-64 | Number treated | 4 |  | 13,850 |  |
| TB preventative therapy for PLHIV | Number treated | 1,128 | 1,128 | 1,800 | Constraint based on number of PLHIV moving from untreated to on ART per year |
| Mass screening | Number screened | 3,977,301 |  |  |  |
| Contact tracing | Number diagnosed | 21 |  | 755 | Number screened=6885 (WHO Global TB Programme, 2023). Constraint on maximum expansion of contact tracing based on: expected number of index cases, allowing for an increase in case-finding; average number of contacts tested per index case (2.8); number of contacts to be tested per index case to meet national targets for contact tracing among child contacts under 5 (6.2); and estimated TB prevalence among household contacts by age group. Sources: [6,7] |
| Active case finding (prisoners) | Total prison population | 31,321 |  | 31,321 | Screening provided to individuals entering the pentitiary system. Estimated 5,417 individuals screened based on the number in pre-trial/remand, which was adjusted to input unit cost per total prison population. Source: [8] |
| Active case finding (PWID) | Number diagnosed | 9 |  | 9 | Number screened through questionnaire (18,284) and X-ray (2,330) based on programme data. Funded through the HIV programme and assumed that current spending and coverage is fixed. |
| Active case finding (community-based) | Number diagnosed | 0 |  | 831 | Prospective intervention, assumed possible to reach 20% of prevalent undiagnosed cases per year. |
| Other testing, including passive case finding | Total population | 9,534,955 |  | 9,534,955 | Estimated coverage of 20,222 people screened adjusted to input unit cost per total population. Constrained maximum coverage to reflect the demand-driven nature of passive case finding. |
| DS-TB treatment | Number treated | 961 |  |  |  |
| MDR-TB treatment (standard) | Number treated | 122 |  |  |  |
| MDR-TB treatment (shorter oral regimens) | Number treated | 501 |  |  |  |
| XDR-TB treatment (standard) | Number treated | 38 |  |  |  |
| XDR-TB treatment (shorter oral regimens) | Number treated | 28 |  |  |  |

|  |  |  |  |  |  |
| --- | --- | --- | --- | --- | --- |
| **KYRGYZ REPUBLIC** | | | | | |
|  |  | **Baseline coverage 2022** | **Minimum constraint *** | **Maximum constraint** | **Notes** |
| BCG vaccination | Number vaccinated | 140,244 | 140,244 |  | Minimum constraint based on maintenance of BCG vaccination through maternal, newborn, and child health programs. |
| TB preventive treatment for 0-4 | Number treated | 215 |  | 1,425 | Constraint determined by National TB Programme based on feasibility for expanded programme coverage [9]. |
| TB preventive treatment for 5-14 | Number treated | 284 |  | 2,296 | Constraint determined by National TB Programme based on feasibility for expanded programme coverage [9]. |
| TB preventive treatment for PLHIV | Number treated | 642 | 642 | 652 | Minimum constraint based on maintenance of preventive treatment for people living with HIV through HIV programs. Maximum constraint applied based on limited opportunities to scale up coverage from the current coverage of 95% of people living with HIV recently diagnosed and initiated on HIV treatment. |
| Household contact tracing | Numbed diagnosed | 271 |  | 358 | Constraint determined by National TB Programme based on feasibility for expanded programme coverage [9]. |
| Active case finding among prisoner populations | Numbed diagnosed | 165 |  | 440 | Constraint determined by National TB Programme based on feasibility for expanded programme coverage [9]. |
| Active case finding among PLHIV | Numbed diagnosed | 275 |  | 353 | Constraint determined by National TB Programme based on feasibility for expanded programme coverage [9]. |
| All other TB testing | Total population | 4,571,603 |  | 6,527,671 | Based on an estimated 4,488 notifications not through active case finding, unit cost was adjusted to represent cost per person in whole population (6,527,671 in 2021) to have testing available, and reflects reduced demand for TB testing services in 2020 and 2021 due to COVID-19-related disruptions at approximately 70% of the 2019 level, with the possibility of returning to 2019 levels of demand in the future |
| DS-TB treatment | Number treated | 3,843 |  |  |  |
| MDR-TB treatment (standard) | Number treated | 689 |  |  |  |
| MDR-TB treatment (shorter oral regimens) | Number treated | 169 |  |  |  |
| XDR-TB treatment (standard) | Number treated | 11 |  |  |  |
| XDR-TB treatment (shorter oral regimens) | Number treated | 0 |  |  |  |

|  |  |  |  |  |  |
| --- | --- | --- | --- | --- | --- |
| **MOLDOVA** | | | | | |
|  |  | **Baseline coverage 2022** | **Minimum constraint *** | **Maximum constraint** | **Notes** |
| BCG vaccination | Number vaccinated | 41,380 | 41,380 |  | Minimum constraint based on maintenance of BCG vaccination through maternal, newborn, and child health programs. |
| TB preventive treatment for 0-4 | Number treated | 81 |  | 113 | Coverage data shown is after adjustment for prevalence of early latent TB. Maximum constraint set based on expected number of index cases, estimated size of household (2.9) and prevalence of early latent TB among household and repeated contacts. Sources: [6,10]. |
| TB preventive treatment for 5-14 | Number treated | 111 |  | 157 |  |
| TB preventive treatment for 15-17 | Number treated | 17 |  | 23 |  |
| TB preventive treatment for 18-64 | Number treated | 80 |  | 373 |  |
| TB preventive treatment for 65+ | Number treated | 0 |  | 86 |  |
| TB preventive treatment for PLHIV | Number treated | 263 | 263 | 263 | Fully constrained |
| Household contact tracing | Number diagnosed | 122 |  | 217 | Maximum number able to be diagnosed derived based on estimated household size (2.9) and active prevalence among contacts. Sources: [6,10] |
| Active case finding (mobile outreach) | Number diagnosed | 378 |  | 756 | Assumes maximum possible coverage for active case finding modalities is double baseline coverage. Actual maximum coverage could be determined based on prioritization combined with feasibility and may be higher or lower. |
| Active case finding (NGO) | Number diagnosed | 172 |  | 344 |  |
| Active case finding (other populations at risk) | Number diagnosed | 1,125 |  | 2,250 |  |
| Passive case finding | Total population | 2,610,000 |  | 2,610,000 | Number of notifications (n=323) adjusted to input cost per person in the country to maintain capacity for on-demand testing. Constrained maximum coverage to reflect the demand-driven nature of passive case finding. |
| DS-TB treatment | Number treated | 1,709 |  |  |  |
| MDR-TB treatment (standard) | Number treated | 251 |  |  |  |
| MDR-TB treatment (shorter oral regimens) | Number treated | 74 |  |  |  |
| XDR-TB treatment (standard) | Number treated | 0 |  |  |  |

|  |  |  |  |  |  |
| --- | --- | --- | --- | --- | --- |
| **TAJIKISTAN** | | | | | |
|  |  | **Baseline coverage 2022** | **Minimum constraint *** | **Maximum constraint** | **Notes** |
| BCG vaccination | Number vaccinated | 247,891 | 247,891 |  | Minimum constraint based on maintenance of BCG vaccination through maternal, newborn, and child health programs. |
| TB preventative therapy for 0-4 | Per person with early latent TB treated | 296 |  |  | Based on coverage of 835 and adjusted for prevalence of early latent TB. |
| TB preventative therapy for 5-14 | Per person with early latent TB treated | 255 |  |  | Based on coverage of 1063 and adjusted for prevalence of early latent TB. |
| TB preventative therapy for 15-64 | Per person with early latent TB treated | 14 |  |  | Based on coverage of 110 and adjusted for prevalence of early latent TB. |
| TB preventative therapy for PLHIV | Number vaccinated | 965 | 965 | 9,230 | Constrained not to reduce below current spending and to not increase beyond the estimated number of PLHIV on ART. |
| Mass screening (school-based) | Number screened | 5,000 |  | 2,378,689 | Constrained based on population size of children 5-14. |
| Mass screening (community TB days) | Number screened | 3000 |  |  |  |
| Contact tracing | Number diagnosed | 281 |  | 1,554 | Constraint on maximum expansion of contact tracing based on: expected number of index cases, allowing for an increase in case-finding; average household size (9) informing number of contacts per index case in Tajikistan study; and estimated TB prevalence among household and repeated contacts by age group. Sources: [6,11]. |
| Active case finding (prisoners) | Total prison population | 12,000 |  | 12,000 | Number x-ray screened (8000) and diagnoses provided by NTP and adjusted to input unit cost per whole prison population. |
| Active case finding (community-based) | Number diagnosed | 625 |  | 5% undiagnosed TB | Based on symptom screening for 33,862 and Xpert testing of 11,529 people, with a yield of 1.8% among those symptom screened. Assumed to be able to reach up 5% of undiagnosed TB cases per year given sufficient funding. |
| Active case finding (hospitals and primary health care) | Total population | 10,278,735 |  | 13,362,355 | Coverage of 2940 adjusted to input unit cost per total population. Assumed 30% increase possible in line with pre-COVID numbers, with the possibility of returning to 2019 levels of demand in the future |
| DS-TB treatment | Number treated | 3,924 |  |  | Treatment coverage values adjusted to reflect only pulmonary TB treatment, while the unit cost reflects total cost for treatment including extra-pulmonary TB |
| MDR-TB standard treatment | Number treated | 238 |  |  |  |
| MDR-TB shorter treatment regimens | Number treated | 153 |  |  |  |
| XDR-TB standard treatment | Number treated | 995 |  |  |  |

|  |  |  |  |  |  |
| --- | --- | --- | --- | --- | --- |
| **UZBEKISTAN** | | | | | |
|  |  | **Baseline coverage 2022** | **Minimum constraint *** | **Maximum constraint** | **Notes** |
| BCG vaccination | Number vaccinated | 728,288 | 728,288 |  | Minimum constraint based on maintenance of BCG vaccination through maternal, newborn, and child health programs. |
| TPT for 0-4 | Per person with early TB reached | 615 |  | 2,441 | Shown baseline coverage and constraint have been adjusted for prevalence of early latent TB. Maximum constraint based on expected number of index cases, average household size (5.24), and estimated TB prevalence among household contacts. Sources: [6,10] |
| TPT for 5-14 | Per person with early TB reached | 1,003 |  | 2,900 |  |
| TPT for 15-64 | Per person with early TB reached | 1,077 |  | 8,395 |  |
| TPT for non-household repeat contacts | Per person with early TB reached | 0 |  | 11,483 | Constraint based on expanded reach of TPT among non-household repeat contacts to enable reach of target of 10 contacts screened per index case when average household size is 5.24. Sources: [10,12] |
| TB preventative therapy for PLHIV | Number treated | 10,512 | 10,512 | 36,488 | Funded through the HIV program and constrained not to reduce below current spending. Maximum possible coverage based on number of PLHIV on ART. |
| Household contact tracing | Number diagnosed | 1,215 |  | 5,679 | Constraint on maximum expansion of contact tracing based on: expected number of index cases, allowing for an increase in case-finding; average household size is 5.24; number of contacts to be tested per index case to meet national targets for contacts tested (n=10); and estimated TB prevalence among household contacts by age group. Sources: [6,10,12] |
| Active case finding (prisoners) | Total prison population | 4,664 |  | 23,320 | Program impact on diagnosis rates assumes prisoners may be screened twice a year based on country input. The number covered and unit cost were inferred based on the diagnosis rate for all prisoners estimated in 2022 (0.2%), the program impact, and total spending. Coverage adjusted to reflect currently eligible population (excluding those already diagnosed and/or on treatment for active TB). |
| Active case finding (people with high risk factors) | Number diagnosed | 1,805 |  | 3,610 | Assumes maximum possible coverage is double baseline coverage. Actual maximum coverage could be determined based on prioritization combined with feasibility and may be higher or lower. |
| Active case finding (mandatory) | Number diagnosed | 694 |  | 1,387 | Assumes maximum possible coverage is double baseline coverage. Actual maximum coverage could be determined based on prioritization combined with feasibility and may be higher or lower. |
| Other testing, including passive case finding and mass screening | Total population | 34,627,591 |  | 34,627,591 | Screening coverage of 2,095,684 adjusted to input cot per total population alive. Constrained maximum coverage to reflect the demand-driven nature of passive case finding. |
| DS-TB treatment | Number treated | 7,878 |  |  |  |
| MDR-TB treatment (standard) | Number treated | 1,366 |  |  |  |
| MDR-TB treatment (shorter oral regimens) | Number treated | 211 |  |  |  |
| XDR-TB treatment (standard) | Number treated | 192 |  |  |  |
| XDR-TB treatment (shorter oral regimens) | Number treated | 93 |  |  |  |

* All programs had a minimum spending constraint of 50% of most recently reported to reflect the challenges of rapid transition of resources.

Table S3. Number of notified TB infections per population group, 2022

| Population group | Sputum positive | | | Sputum negative | | | Total notified |
| --- | --- | --- | --- | --- | --- | --- | --- |
|  | DS-TB | MDR-TB | XDR-TB | DS-TB | MDR-TB | XDR-TB |  |
| BELARUS |  |  |  |  |  |  |  |
| 0-4 | 0 | 0 | 0 | 0 | 0 | 0 | 0 |
| 5-14 | 1 | 0 | 0 | 3 | 0 | 0 | 4 |
| 15-64 | 723 | 618 | 17 | 93 | 54 | 0 | 1505 |
| 65+ | 177 | 86 | 1 | 29 | 7 | 0 | 300 |
| Prisoners | 17 | 34 | 0 | 0 | 3 | 0 | 54 |
| PLHIV not on ART | 2 | 5 | 0 | 0 | 1 | 0 | 8 |
| PLHIV on ART | 40 | 69 | 2 | 3 | 5 | 0 | 119 |
| Total | 960 | 812 | 20 | 128 | 70 | 0 | 1990 |
| **KYRGYZ REPUBLIC** |  |  |  |  |  |  |  |
| 0-4 | 19 | 16 | 1 | 32 | 4 | 0 | 72 |
| 5-14 | 67 | 39 | 1 | 115 | 9 | 0 | 231 |
| 15-64 | 911 | 568 | 16 | 1668 | 137 | 2 | 3302 |
| 65+ | 189 | 70 | 2 | 330 | 17 | 0 | 608 |
| Prisoners | 17 | 8 | 0 | 7 | 2 | 0 | 34 |
| Total | 1203 | 701 | 20 | 2152 | 169 | 2 | 4247 |
| **MOLDOVA** |  |  |  |  |  |  |  |
| 0-4 | 0 | 0 | 0 | 30 | 5 | 0 | 35 |
| 5-14 | 2 | 0 | 0 | 40 | 6 | 0 | 48 |
| 15-17 | 2 | 2 | 0 | 10 | 8 | 0 | 22 |
| 17-64 | 627 | 253 | 0 | 889 | 188 | 0 | 1,957 |
| 65+ | 59 | 11 | 0 | 123 | 3 | 0 | 196 |
| PLHIV, 0-4 | 0 | 0 | 0 | 2 | 0 | 0 | 2 |
| PLHIV, 5-14 | 0 | 0 | 0 | 1 | 0 | 0 | 1 |
| PLHIV, 15-17 | 0 | 0 | 0 | 0 | 0 | 0 | 0 |
| PLHIV, 18-64 | 67 | 35 | 0 | 119 | 36 | 0 | 257 |
| PLHIV, 65+ | 1 | 0 | 0 | 3 | 0 | 0 | 4 |
| Prisoners | 16 | 6 | 0 | 31 | 13 | 0 | 66 |
| Migrants | 97 | 42 | 0 | 102 | 21 | 0 | 262 |
| Total | 871 | 349 | 0 | 1,350 | 280 | 0 | 2,850 |
| **TAJIKISTAN** |  |  |  |  |  |  |  |
| 0-4 | 61 | 15 | 0 |  |  |  | 76 |
| 5-14 | 160 | 14 | 2 |  |  |  | 176 |
| 15-64 | 2017 | 193 | 4 |  |  |  | 2,214 |
| 65+ | 420 | 27 | 2 |  |  |  | 449 |
| Prisoners | 48 | 23 | 1 |  |  |  | 72 |
| Migrant workers | 752 | 38 | 1 |  |  |  | 791 |
| PLHIV not on ART | 69 | 4 | 0 |  |  |  | 73 |
| PLHIV on ART | 69 | 4 | 0 |  |  |  | 73 |
| Total | 3,597 | 317 | 10 |  |  |  | 3,924 |
| **UZBEKISTAN** |  |  |  |  |  |  |  |
| 0-4 | 5 | 0 | 0 | 279 | 7 | 6 | 297 |
| 5-14 | 50 | 22 | 2 | 1037 | 61 | 21 | 1,193 |
| 15-64 | 3720 | 1125 | 174 | 4929 | 46 | 22 | 10,016 |
| 65+ | 1602 | 211 | 19 | 1016 | 5 | 0 | 2,853 |
| Prisoners | 23 | 20 | 9 | 18 | 0 | 1 | 71 |
| PLHIV not on ART | 39 | 17 | 2 | 70 | 0 | 0 | 128 |
| PLHIV on ART | 87 | 64 | 11 | 149 | 1 | 0 | 312 |
| Total | 5,526 | 1,459 | 217 | 7,498 | 120 | 50 | 14,870 |

Notes: ART, antiretroviral therapy; DS, drug susceptible; MDR, multi-drug resistant; PLHIV, people living with HIV; TB, tuberculosis; XDR, extensively drug-resistant. Sputum smear status not differentiated in Tajikistan.

Sources: National TP Programme data from participating countries, 2023

Table S4. Demographic input data, 2022

| Parameter | Belarus | Kyrgyz Republic | Moldova | Tajikistan | Uzbekistan |
| --- | --- | --- | --- | --- | --- |
| Population size |  |  |  |  |  |
| 0-4 | 452,324 | 797,905 | 208,892 | 1,269,089 | 3,911,385 |
| 5-14 | 1,147,246 | 1,486,085 | 422,858 | 2,340,744 | 6,550,093 |
| 15-64 | 6,238,536 | 4,039,463 | 1,982,034 | 5,509,606 | 22,286,198 |
| 65+ | 1,638,528 | 301,146 | 423,889 | 345,556 | 1,778,596 |
| Prisoners | 31,321 | 6,000 | 6,315 | 12,000 | 23,320 |
| PLHIV not on ART | 5,9182 |  | 23,458 | 7,897 | 41,512 |
| PLHIV on ART | 21,082 |  |  | 5,103 | 36,488 |
| Migrant workers |  |  | 92,190 | 461,757 |  |
| Percentage of people who age into the next age category per year | | | | | |
| 0-4 | 21% | 20% | 201% | 20% | 19% |
| 5-14 | 9% | 8% | 9% | 9% | 9% |
| 15-64 | 2% | 1% | 34%, 2%^a^ | 1% | 1% |
| Annual number of births | 87,541 | 154,510 | 43,678 | 258,555 | 776,815 |
| Annual non-TB death rate | | | | | |
| 0-4 | 0.05% | 0.29% | 0.26% | 0.6% | 0.3% |
| 5-14 | 0.01% | 0.02% | 0.02% | 0.0% | 0.0% |
| 15-64 | 0.58% | 0.43% | 0.03%, 0.72%^a^ | 0.3% | 0.4% |
| 65+ | 6.73% | 6.54% | 8.15% | 5.8% | 6.2% |
| Prisoners | 2.1% | 0.36% | 1.03% | 0.6% | 0.9% |
| PLHIV not on ART | 0.58% |  | 0.02%-8.15%^b^ | 0.3% | 0.4% |
| PLHIV on ART | 0.58% |  |  | 0.3% | 0.4% |
| Migrant workers |  |  | 1.03% | 0.3% |  |
| Net number of migrants | 30,308 | -10,000 | 390,000 | 19,999 | -19,999 |

Source: UN Population Division 2022 [13]. Additional data on prisoners, migrants and people living with HIV from World Prison Brief, Asian Development Bank [14], UNAIDS spectrum estimates [15] and country-provided estimates.

Notes: All-cause mortality adjusted during calibration. Total number of migrants distributed by age-weighting. a, 15-17 and 18-64 years modelled; b, People living with HIV modelled for all age groups.

Table S5. Epidemiological parameters by country model

| **Description** | **Population** | **Belarus** | **Kyrgyz Republic** | **Moldova** | **Tajikistan** | **Uzbekistan** | **Source or assumptions** |
| --- | --- | --- | --- | --- | --- | --- | --- |
| Early Latency Departure Rate | 0-4 | 0.2 | 0.2 | 0.2 | 0.2 | 0.2 | Houben, Lalli (2016) - appendix of TIME model. 0.1%/year reactivation rate (0.01-0.25). |
|  | 5-14 | 0.2 | 0.2 | 0.2 | 0.2 | 0.2 |  |
|  | 15-64 | 0.2 | 0.2-0.26 | 0.2 | 0.2 | 0.2 |  |
|  | 65+ | 0.2 | 0.3-0.39 | 0.2 | 0.2 | 0.3 |  |
|  | Prisoners | 0.2 | 0.25 | 0.5 | 0.2 | 0.3 |  |
|  | PLHIV on ART | 0.2 | N/A | 2.0-4.0 | 0.2 | 0.3 |  |
|  | PLHIV not on ART | 0.99 | N/A |  | 0.99 | 0.5 |  |
|  | Migrant workers | N/A | N/A | 0.3 | 0.2 | N/A |  |
| Late Latency Departure Rate | 0-4 | 0.001 | 0.003 | 0.002 | 0.0015-0.006 | 0.0001 | Andrews, Noubary (2012) - risk of progression to active. Where range reported, assumed decrease in late latency departure rate over time. |
|  | 5-14 | 0.001 | 0.003 | 0.0005 | 0.0005-0.002 | 0.0003 |  |
|  | 15-64 | 0.0002-0.00135 | 0.003 | 0.0007-0.0015 | 0.0005-0.002 | 0.0005 |  |
|  | 65+ | 0.0005-0.003 | 0.003 | 0.0007 | 0.0005-0.006 | 0.0035 |  |
|  | Prisoners | 0.003 | 0.0032 | 0.01-0.3 | 0.0015-0.006 | 0.0035 |  |
|  | PLHIV not on ART | 0.007 | N/A | 0.1 | 0.05-0.2 | 0.1 |  |
|  | PLHIV on ART | 0.0009-0.007 | N/A |  | 0.0015-0.006 | 0.001 |  |
|  | Migrant workers | N/A | N/A | 0.003 | 0.0015-0.006 | N/A |  |
| Probability of Early-Active vs. Early-Late LTBI Progression | 0-4 | 0.177 | 0.2 | 0.177 | 0.19 | 0.1 | Andrews, Noubary (2012) - risk of progression to active. Where range reported, assumed decrease in LTBI progression over time. |
|  | 5-14 | 0.177 | 0.15 | 0.177 | 0.17 | 0.09 |  |
|  | 15-64 | 0.177 | 0.18-0.23 | 0.177 | 0.12 | 0.2 |  |
|  | 65+ | 0.177 | 0.2 | 0.177 | 0.3 | 0.354 |  |
|  | Prisoners | 0.1947 | 0.25 | 0.531 | 0.17 | 0.531 |  |
|  | PLHIV not on ART | 0.93 | N/A | 0.93 | 0.93 | 0.7 |  |
|  | PLHIV on ART | 0.177-0.354 | N/A |  | 0.17 | 0.6 |  |
|  | Migrant workers | N/A | N/A | 0.531 | 0.17 | N/A |  |
| Infection Vulnerability Factor (Vaccinated vs. Susceptible) | 0-14 | 0.5 | 0.5 | 0.5 | 0.5 | 0.5 | Mangtani, Abubakar (2014) - protective efficacy of BCG found to range from 0-80%. |
|  | 15+ | 1.0 | 1.0 | 1.0 | 1.0 | 1.0 |  |
| Infection vulnerability factor (relative population susceptibility) | 0-4 | 3.6 | 1.5-1.8 | 3-6 | 5.6 | 2.2 | A value of '1' is the default, but this is likely to be significantly higher in vulnerable populations such as people living with HIV. |
|  | 5-14 | 1.9 | 2.4-2.88 | 0.3 | 2.0 | 2.8 |  |
|  | 15-64 | 1.9 | 2.1-4.2 | 2.0-8.0 | 2.16 | 2.8 |  |
|  | 65+ | 6.5 | 3.0-9.0 | 2.0 | 3.2 | 4.5-5.0 |  |
|  | Prisoners | 7.5 | 30 | 6.0-12.0 | 11 | 5.0-6.0 |  |
|  | PLHIV not on ART | 24 | N/A | 1.0-16.0 | 24 | 2.3 |  |
|  | PLHIV on ART | 10 | N/A |  | 3.2 | 5.5-11.0 |  |
|  | Migrant workers | N/A | N/A | 2.1-6 | 5.6 | N/A |  |
| SP-TB infectiousness | 0-4 | 1.0 | 1.0 | 1.0 | 0.3 | 0.5 | A value of '1' is the default |
|  | 5-14 | 1.0 | 1.0 | 1.0 | 0.7 | 1.0 |  |
|  | 15-64 | 1.0 | 1.0 | 1.0 | 1.0 | 1.0 |  |
|  | 65+ | 1.0 | 1.0 | 1.0 | 1.0 | 1.0 |  |
|  | Prisoners | 5.0 | 1.0 | 1.0 | 1.0 | 1.0 |  |
|  | PLHIV not on ART | 1.0 | N/A | 0.66 | 1.0 | 1.0 |  |
|  | PLHIV on ART | 1.0 | N/A | 0.66 | 1.0 | 1.0 |  |
|  | Migrant workers | N/A | N/A | 1.0 | 1.0 | N/A |  |
| SN-TB infectiousness (compared to SP-TB) | All populations | 0.22 | 0.22 | 0.22 | 0.22 | 0.22 | Behr (1999) |
| Duration of active TB until natural outcome (years) | 0-4 | 3.5 | 3.5 | 3.5 | 3.5 | 3.5 | Glaziou, Sismanidis (2016), Tiemersma, van der Werf (2011) |
|  | 5-14 | 3.5 | 3.5 | 3.5 | 3.5 | 3.5 |  |
|  | 15-64 | 3.5 | 3.0-3.5 | 3.5 | 3.5 | 3.5 |  |
|  | 65+ | 3.5 | 2.5-3.0 | 3.5 | 3.5 | 3.5 |  |
|  | PLHIV not on ART | 2.0 | N/A | 2.0 | 2.0 | 2.0 |  |
|  | PLHIV on ART | 3.5 | N/A |  | 3.5 | 3.5 |  |
|  | Migrant workers | N/A | N/A | 3.5 | 3.5 | N/A |  |
| SP untreated-TB death rate | 0-4 | 30-80% | 24-40% | 35% | 14-19% | 43-86% | Glaziou, Sismanidis (2016), Tiemersma, van der Werf (2011). Where range reported, assumed that death rate decreased over time. For Tajikistan, overall untreated-TB death rate shown, calibrated to align with national reported TB mortality rates given an implied ratio of SP to SN. |
|  | 5-14 | 30-80% | 12-20% | 35% | 14-19% | 35-70% |  |
|  | 15-64 | 30-80% | 12-20% | 35% | 24-37% | 35-70% |  |
|  | 65+ | 30-80% | 18-30% | 35% | 24-37% | 43-86% |  |
|  | Prisoners | 30-80% | 12-20% | 35% | 24-37% | 35-70% |  |
|  | PLHIV not on ART | 83% | N/A | 83% | 67-95% | 90% |  |
|  | PLHIV on ART | 83% | N/A |  | 48-75% | 35-70% |  |
|  | Migrant workers | N/A | N/A | 35% | 24-37% | N/A |  |
| SN untreated-TB death rate | 0-4 | 12-30% | 2.0-3.3% | 10% | N/A | 15-30% | Glaziou, Sismanidis (2016), Tiemersma, van der Werf (2011). Where range reported, assumed that death rate decreased over time. |
|  | 5-14 | 12-30% | 2.0-3.3% | 10% | N/A | 10-20% |  |
|  | 15-64 | 12-30% | 2.0-3.3% | 10% | N/A | 10-20% |  |
|  | 65+ | 12-30% | 4.0-6.7% | 10% | N/A | 10-20% |  |
|  | Prisoners | 12-30% | 2.0-3.3% | 10% | N/A | 10-20% |  |
|  | PLHIV not on ART | 74% | N/A | 74% | N/A | 30% |  |
|  | PLHIV on ART | 12-30% | N/A |  | N/A | 10-20% |  |
|  | Migrant workers | N/A | N/A | 10% | N/A | N/A |  |

Notes: ART, antiretroviral therapy; LTBI, latent TB infection; N/A, not applicable (not modelled); PLHIV, people living with HIV; TB, tuberculosis. Sputum smear status not differentiated in Tajikistan.

**References**

1. National TB Protection Program of the Republic of Tajikistan, Burnet Institute, Global Fund. Evaluating cost-effective investments to reduce the burden of drug-resistant tuberculosis (TB) in Tajikistan: Findings from an Optima TB analysis, 2023. Melbourne, Australia: Burnet Institute; 2024. Available from: <https://optimamodel.com/pubs/OptimaTB_Tajikistan2023_FinalReport.pdf>.

2. National Tuberculosis Control Program, Burnet Institute, Global Fund. Evaluating cost-effective investments to reduce the burden of drug-resistant tuberculosis (TB) in Uzbekistan: Findings from an Optima TB analysis, 2023. Melbourne, Australia: Burnet Institute; 2024. Available from: <https://optimamodel.com/pubs/Uzbekistan_TB_2024.pdf>.

3. National Tuberculosis Control Program Kyrgyz Republic, Burnet Institute, Global Fund. Evaluating cost-effective investments to reduce the burden of drug-resistant tuberculosis (TB) in Kyrgyz Republic: Findings from an Optima TB analysis, 2023. Melbourne, Australia: Burnet Institute; 2024. Available from: <https://optimamodel.com/pubs/OptimaTB_Kyrgyzstan_2023FinalReport_v3.0_20240313.pdf>.

4. National Tuberculosis Response Program, Burnet Institute, Global Fund. Evaluating cost-effective investments to reduce the burden of drug-resistant tuberculosis (TB) in Moldova: Findings from an Optima TB analysis, 2023. Melbourne, Australia: Burnet Institute; 2024. Available from: <https://optimamodel.com/pubs/Moldova_TB_2024.pdf>.

5. Republican Research and Practical Centre for Pulmonology and Tuberculosis, Burnet Institute, Global Fund. Evaluating cost-effective investments to reduce the burden of drug-resistant tuberculosis (TB) in Belarus: Findings from an Optima TB analysis, 2023. Melbourne, Australia: Burnet Institute; 2024. Available from: <https://optimamodel.com/pubs/Belarus_TB_2024.pdf>.

6. Fox GJ, Barry SE, Britton WJ, Marks GB. Contact investigation for tuberculosis: a systematic review and meta-analysis. European Respiratory Journal. 2013;41(1):140-56.

7. Ministry of Health Republic of Belarus. Strategic plan for TB control in Republic of Belarus for 2021-2025. Unpublished report. Received 2023, July 20.; 2021.

8. World Prison Brief: Belarus: Institute for Crime & Justic Policy Research; [cited 2023 Aug 8]. Available from: <https://www.prisonstudies.org/country/belarus>.

9. Optimizing Investments in Kyrgyz Republic’s Tuberculosis Response: Results of a TB Allocative Efficiency Study. Washington DC: World Bank; 2021.

10. Global Tuberculosis Programme Data: World Health Organization; 2023 [updated Nov 7; cited 2023 Nov 9]. Available from: <https://www.who.int/teams/global-tuberculosis-programme/data>.

11. Rekart M, Aung A, Cullip T, Mulanda W, Mun L, Pirmahmadzoda B, et al. Household drug-resistant TB contact tracing in Tajikistan. The International Journal of Tuberculosis and Lung Disease. 2023;27(10):748-53.

12. World Health Organization Regional Office for Europe. Extensive review of the National Tuberculosis Programme in the Republic of Uzbekistan: 3–19 October 2022. Copenhagen; 2022.

13. United Nations Department of Economic and Social Affairs Population Division. 2022 Revision of World Population Prospects 2022 [cited 2022 July 23]. Available from: <https://population.un.org/wpp/>.

14. Strengthening support for labor migration in Tajikistan: assessment and recommendations. Manila: Asian Development Bank; 2020 [cited 2022 Sep 12]. Available from: <https://www.adb.org/sites/default/files/publication/681666/support-labor-migration-tajikistan.pdf>.

15. HIV estimates with uncertainty bounds 1990-present [Internet]. UNAIDS. 2023 [cited 2023 Jul 17]. Available from: <https://www.unaids.org/en/resources/documents/2023/HIV_estimates_with_uncertainty_bounds_1990-present>.

16. Houben RMGJ, Lalli M, Sumner T, Hamilton M, Pedrazzoli D, Bonsu F, et al. TIME Impact – a new user-friendly tuberculosis (TB) model to inform TB policy decisions. BMC Medicine. 2016;14(1):56.

17. Andrews JR, Noubary F, Walensky RP, Cerda R, Losina E, Horsburgh CR. Risk of progression to active tuberculosis following reinfection with Mycobacterium tuberculosis. Clin Infect Dis. 2012;54(6):784-91.

18. Mangtani P, Abubakar I, Ariti C, Beynon R, Pimpin L, Fine PE, et al. Protection by BCG vaccine against tuberculosis: a systematic review of randomized controlled trials. Clin Infect Dis. 2014;58(4):470-80.

19. Glaziou P, Sismanidis C, Zignol M, Floyd K. Methods used by WHO to estimate the global burden of TB disease. Global TB Programme. Geneva: World Health Organization; 2016 [cited 2017 Jan 26]. Available from: <https://www.who.int/tb/publications/global_report/gtbr2016_online_technical_appendix_global_disease_burden_estimation.pdf>.

20. Tiemersma EW, van der Werf MJ, Borgdorff MW, Williams BG, Nagelkerke NJ. Natural history of tuberculosis: duration and fatality of untreated pulmonary tuberculosis in HIV negative patients: a systematic review. PLoS One. 2011;6(4):e17601.
