# Supplementary material for: Optimising TB investments in Belarus, Moldova, Kyrgyz Republic, Tajikistan and Uzbekistan: An allocative efficiency analysis
Source: PLOS Glob Public Health. 2025 Jul 11;5(7):e0004548. doi: 10.1371/journal.pgph.0004548 (PMC12250568; doi:10.1371/journal.pgph.0004548)
Supplement: S3 Text — (DOCX) [file pgph.0004548.s003.docx]

# S3. Calibration methods and figures

**Calibration methods**

Optima TB was calibrated to national epidemiologic data on TB case notifications and WHO estimated TB incidence (Global TB Programme 2022 estimates) through a consultative and iterative approach with country teams, accounting for the reliability of different data sources.

Specific adjusted model parameters by population included:

- Initialization values in 2000
- Relative population latent TB infection susceptibility, as the key calibration parameter
- Latent TB activation rates, reflecting changes over time in population health, especially for people living with HIV with changing HIV treatment coverage
- TB mortality rates for people with untreated TB assuming an average active TB duration of 3.5 years, reflecting increases in diagnosis rates for people with symptomatic TB and hence reductions in the proportion of people with active TB who die prior to diagnosis or initiating treatment, to align with national estimates of TB-related mortality.

Uncertainty ranges in model output were generated through sampling of model parameters within uncertainty ranges corresponding to ±10% of the highest historic value by population (all initialization values, relative population latent TB susceptibility, untreated TB progression rates including progression from latent to active TB, and proportions of new infections by smear/strain). 100 parameter sets were run across scenarios to generate a range of epidemic outcomes, and interquartile range of projections used to represent uncertainty intervals as these most closely reflected WHO uncertainty intervals for data used in calibration. The most important assumptions in the optimization analysis are associated with the cost-coverage and coverage-outcome curves.

**Model optimization weightings**

Allocations were optimised to evenly prioritize progress by 2030 on proportionally reducing each of the prevalence of DS-TB, MDR-TB and XDR-TB and reducing the number of TB-related deaths (Belarus and Moldova considered MDR-TB and XDR-TB together as a single category of DR-TB).

Table A. Model optimization weightings

|  | **DS-TB** | **MDR-TB** | **XDR-TB** | **TB-related death** |
| --- | --- | --- | --- | --- |
| Belarus | 1 | 1 | 1 | 8 |
| Kyrgyz Republic | 1 | 2 | 30 | 25 |
| Moldova | 1 | 3 | 3 | 11 |
| Tajikistan | 1 | 6 | 72 | 11 |
| Uzbekistan | 1 | 3 | 20 | 11 |

Notes: DS, drug susceptible; MDR, multi-drug resistant XDR, extensively drug-resistant; TB, tuberculosis.

Sources: Optima TB country models, 2023

**Selected calibration figures by country**

| **BELARUS** |  |
| --- | --- |
| **TB Incidence – total cases**  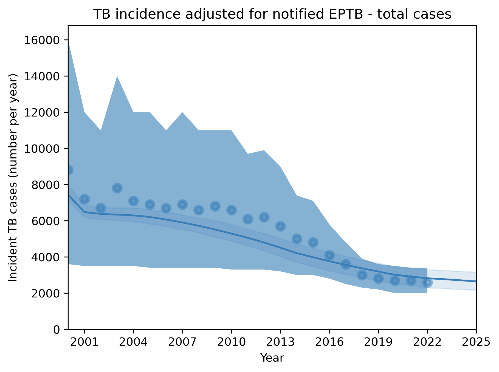 | **Incidence of TB per 100K – total**  **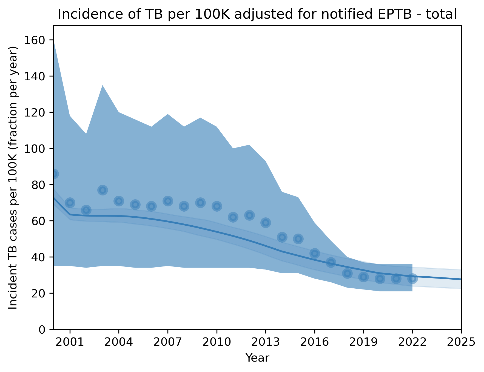** |
| **DR-TB incidence – total cases**  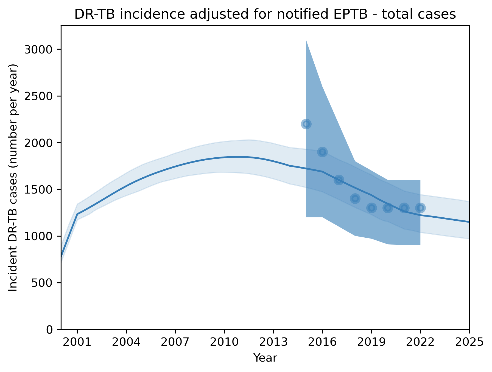 | **Latent TB prevalence – total**  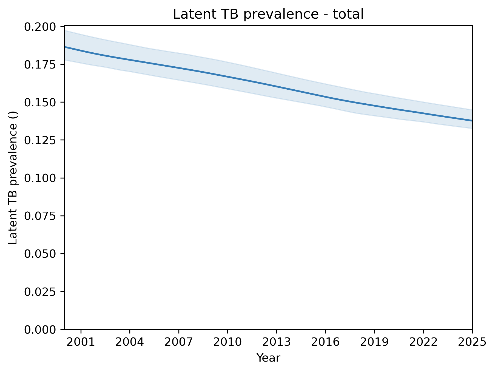 |
| **TB cases notification rate– total**  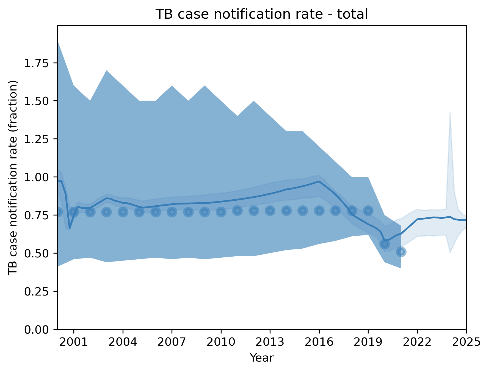 | **TB-related deaths – total**  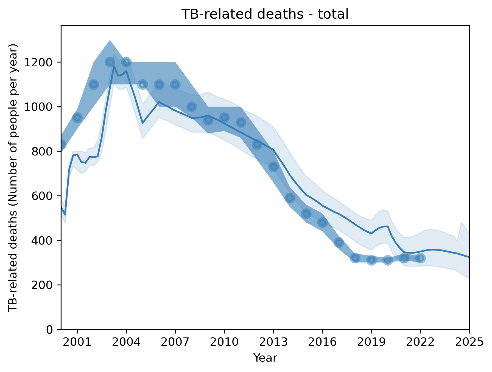 |
| **Case fatality ratio – pulmonary TB total**  **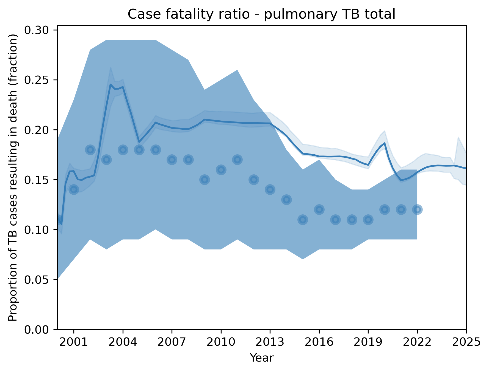** | **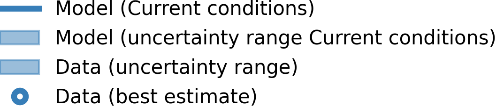** |
| **Cumulative TB incidence 2024 to 2030**  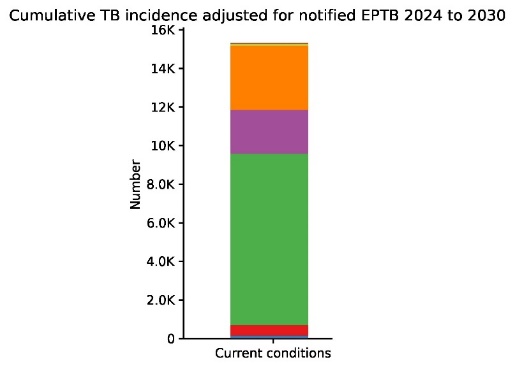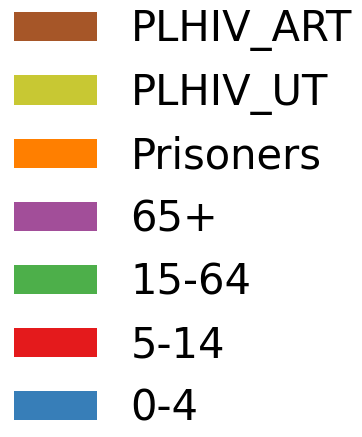 | **Cumulative TB-related deaths 2024 to 2030**  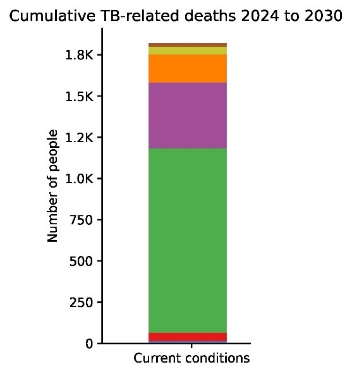 |

| **Kyrgyz Republic** |  |
| --- | --- |
| **TB Incidence – total cases**  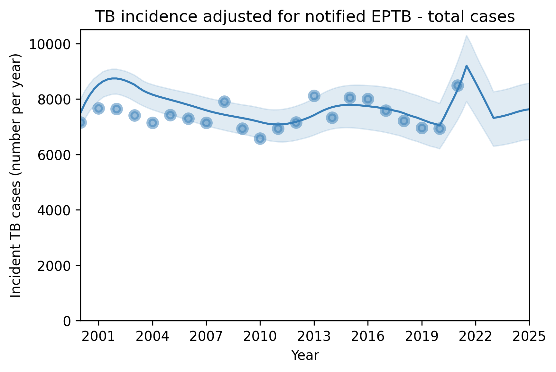 | **Incidence of TB per 100K – total**  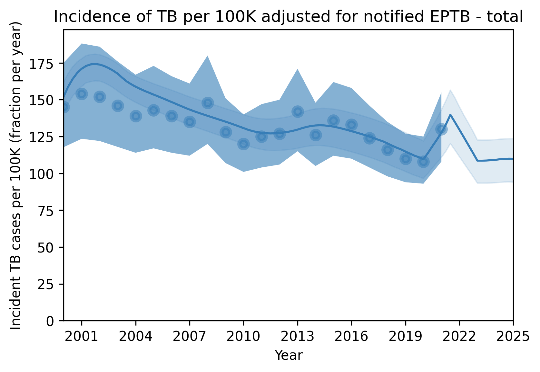 |
| **DR-TB incidence – total cases**  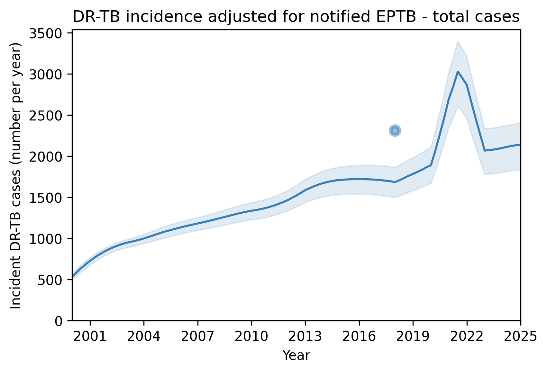 | **Latent TB prevalence – total**  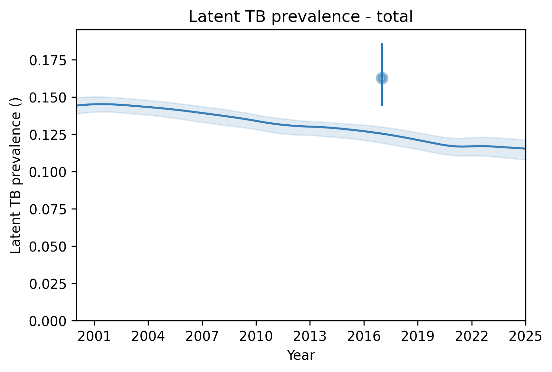 |
| **TB cases notification rate– total**  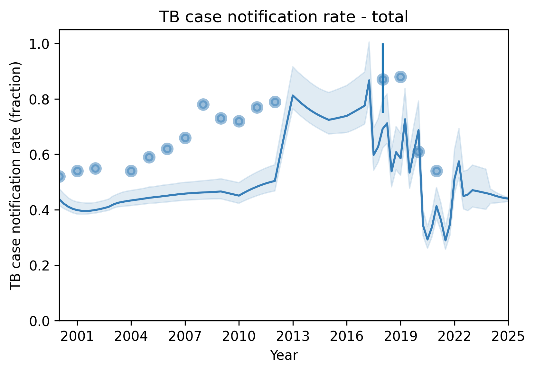 | **TB-related deaths – total**  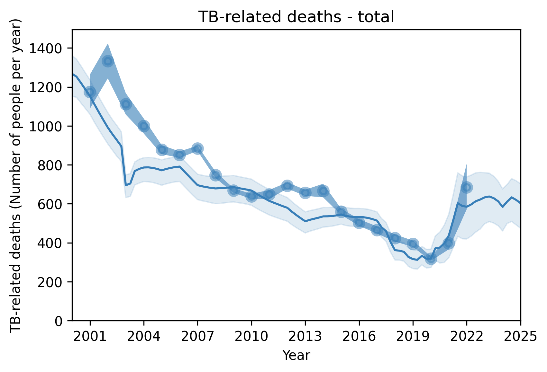 |
| **Case fatality ratio – pulmonary TB total**  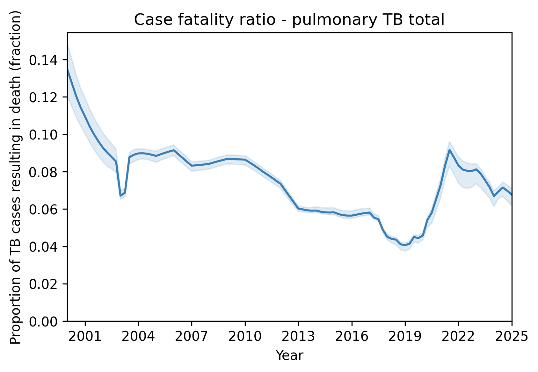 | **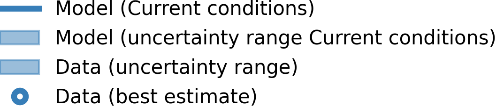** |
| **Cumulative TB incidence 2024 to 2030**  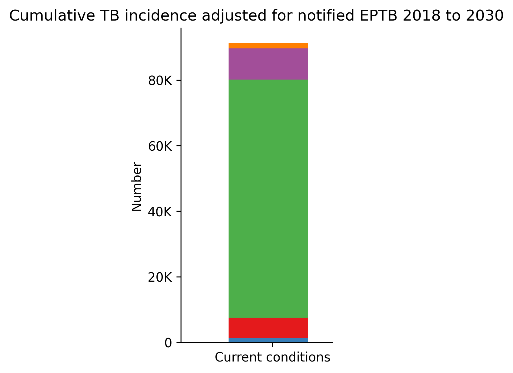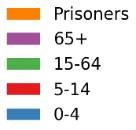 | **Cumulative TB-related deaths 2024 to 2030**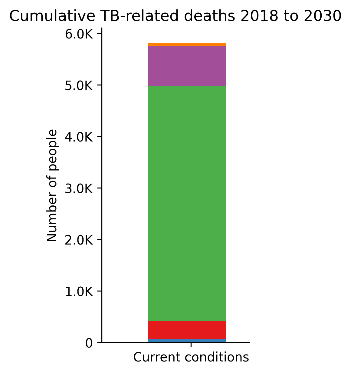 |

| **Moldova** |  |
| --- | --- |
| **TB Incidence – total cases**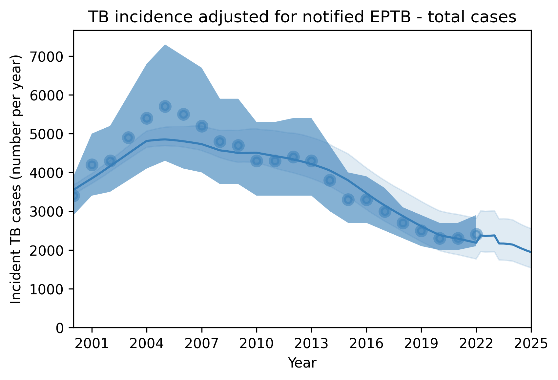 | **Incidence of TB per 100K – total**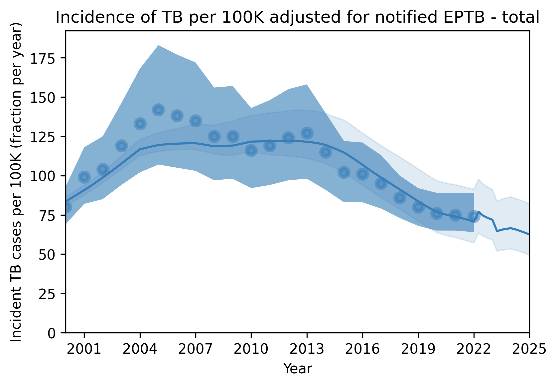 |
| **DR-TB incidence – total cases**  **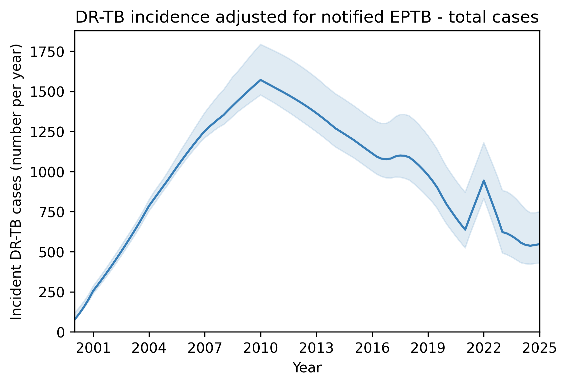** | **Latent TB prevalence – total**  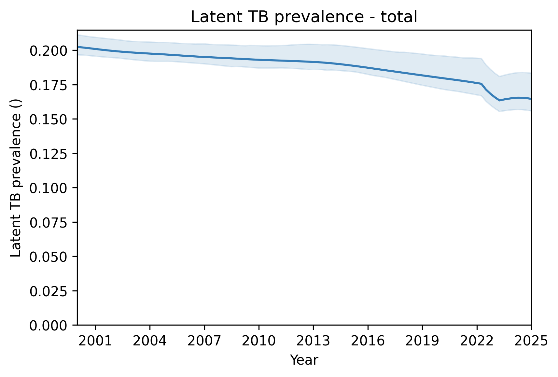 |
| **TB cases notification rate– total**  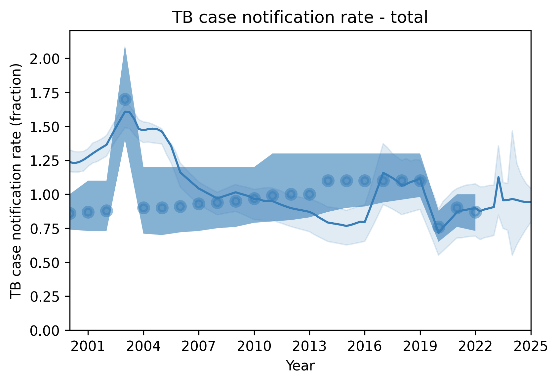 | **TB-related deaths – total**  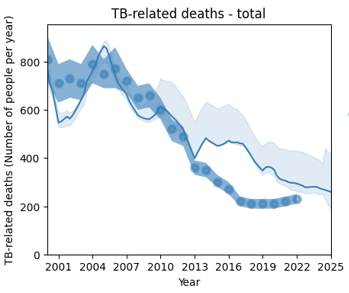 |
| **Case fatality ratio – pulmonary TB total**  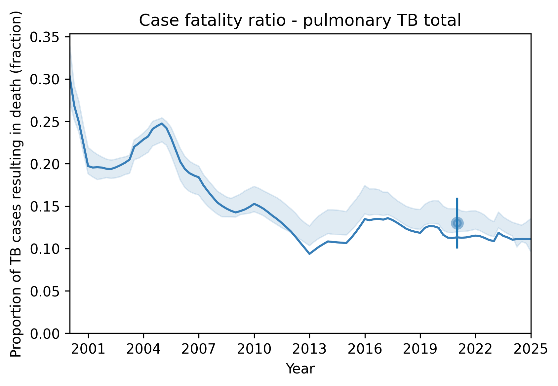 | **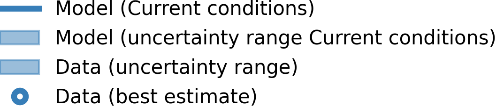** |
| **Cumulative TB incidence 2024 to 2030**  **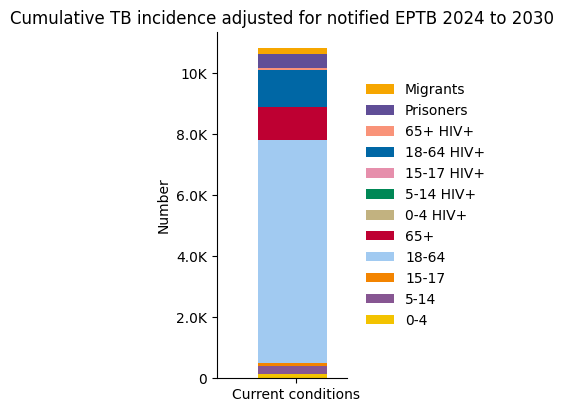** | **Cumulative TB-related deaths 2024 to 2030**  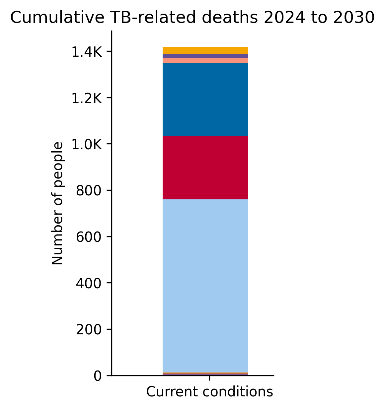 |

| **Tajikistan** |  |
| --- | --- |
| **TB Incidence – total cases**  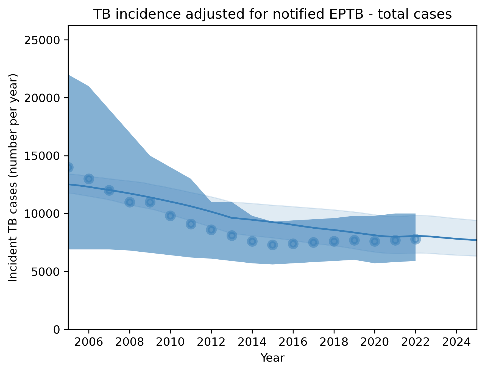 | **Incidence of TB per 100K – total**  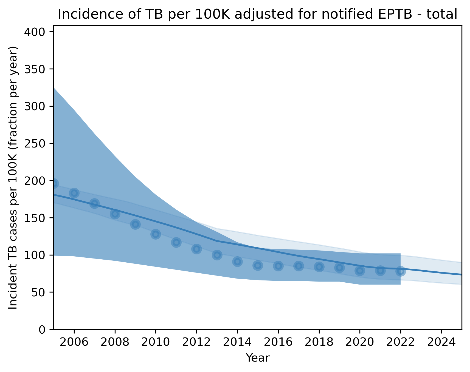 |
| **DR-TB incidence – total cases**  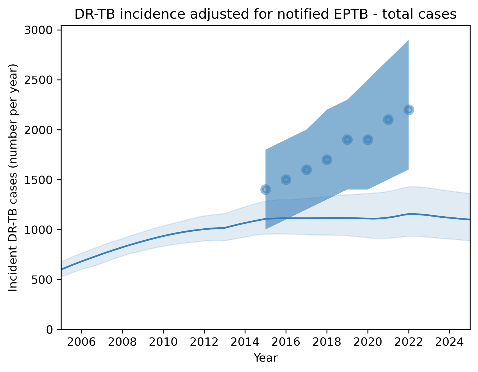 | **Latent TB prevalence – total**  **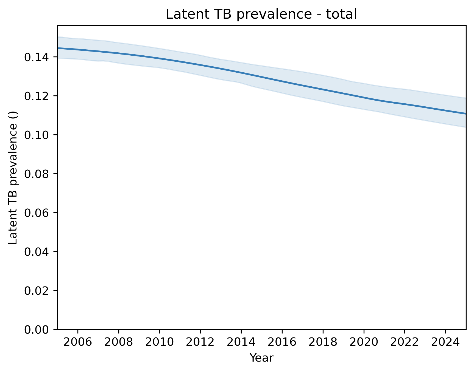** |
| **TB cases notification rate– total**  **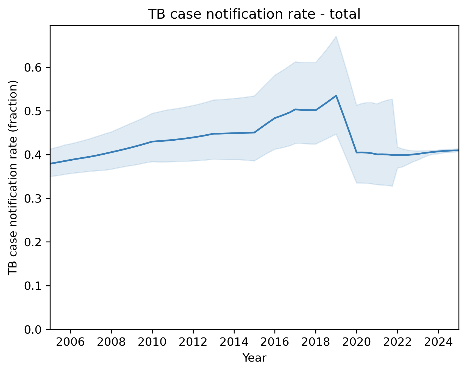** | **TB-related deaths – total**  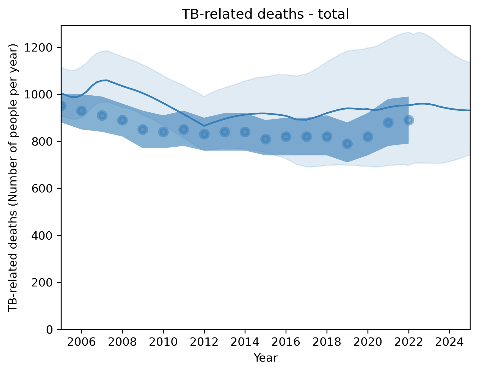 |
| **Case fatality ratio – pulmonary TB total**  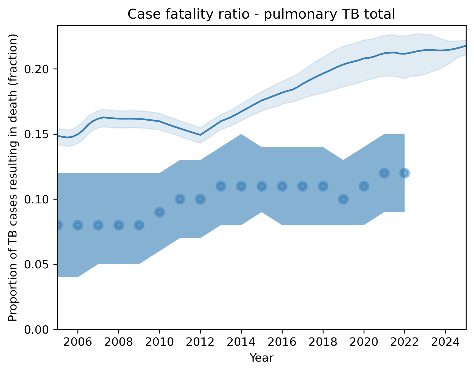 | **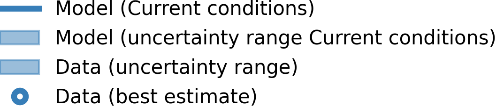** |
| **Cumulative TB incidence 2024 to 2030** 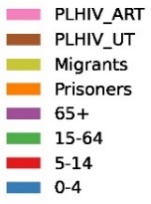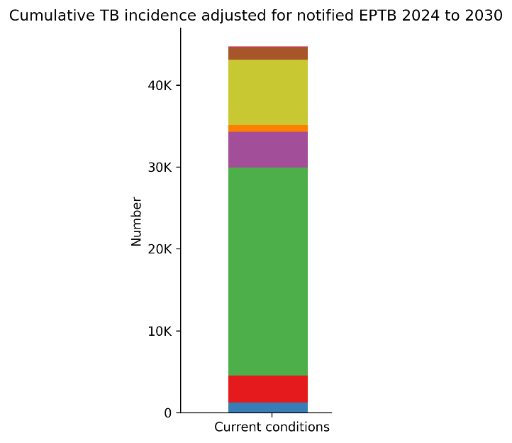 | **Cumulative TB-related deaths 2024 to 2030**  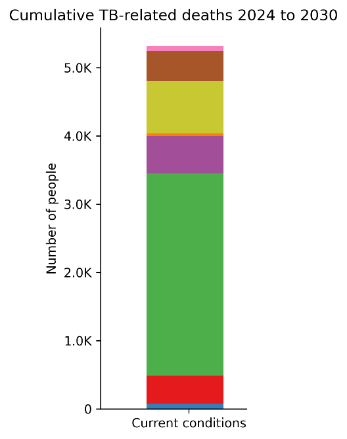 |

| **Uzbekistan** |  |
| --- | --- |
| **TB Incidence – total cases**  **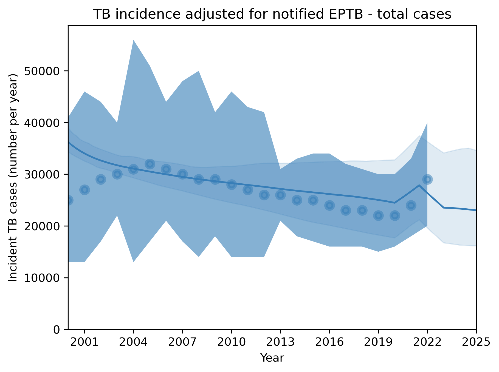** | **Incidence of TB per 100K – total**  **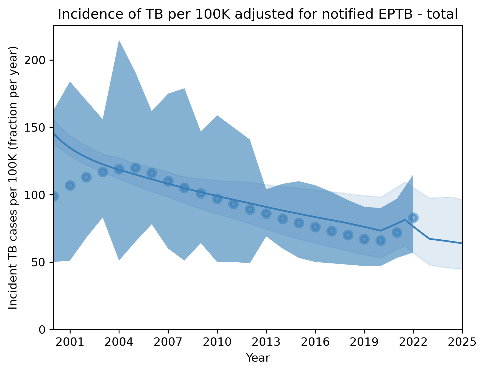** |
| **DR-TB incidence – total cases**  **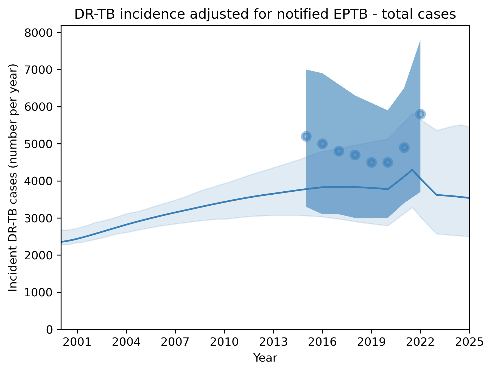** | **Latent TB prevalence – total**  **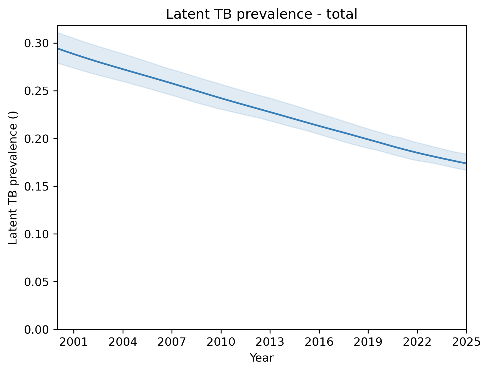** |
| **TB cases notification rate– total**  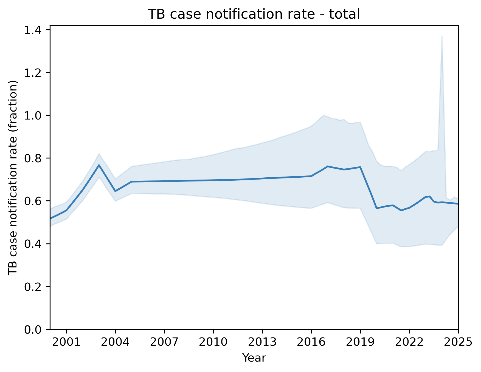 | **TB-related deaths – total**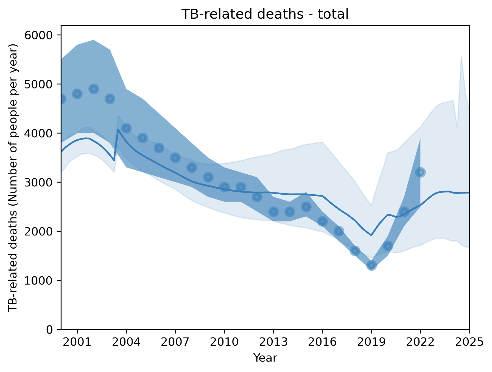 |
| **Case fatality ratio – pulmonary TB total**  **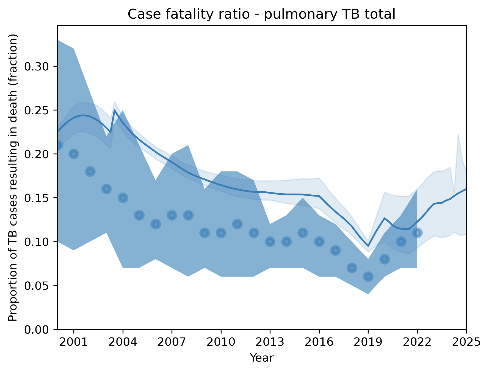** | **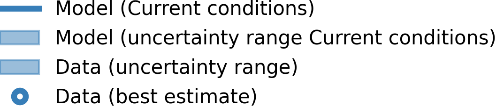** |
| **Cumulative TB incidence 2024 to 2030** 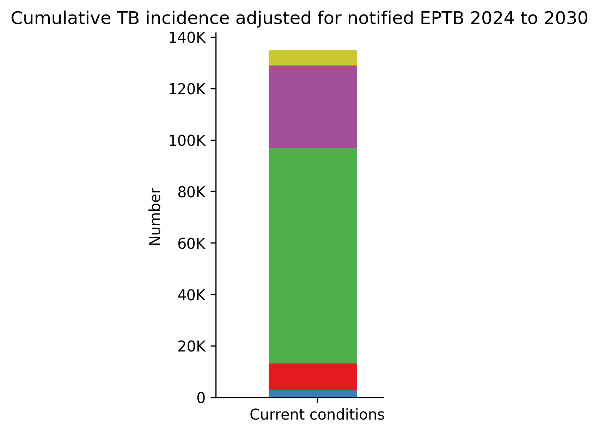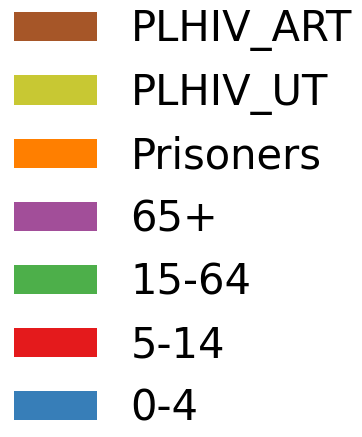 | **Cumulative TB-related deaths 2024 to 2030**  **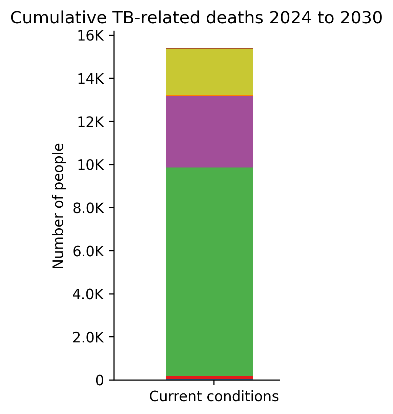** |
