# Supplementary material for: Optimising TB investments in Belarus, Moldova, Kyrgyz Republic, Tajikistan and Uzbekistan: An allocative efficiency analysis
Source: PLOS Glob Public Health. 2025 Jul 11;5(7):e0004548. doi: 10.1371/journal.pgph.0004548 (PMC12250568; doi:10.1371/journal.pgph.0004548)
Supplement: S4 Text — (DOCX) [file pgph.0004548.s004.docx]

# S4. Detailed findings by country

**Optimised spending allocation in baseline spending (2022), optimised spending 100% and optimised spending 150%**

Table A. Spending allocations in baseline and optimised spending 100% and optimised spending 150% scenarios

|  | **Belarus** | | | **Kyrgyz Republic** | | | **Moldova** | | | **Tajikistan** | | | **Uzbekistan** | | |
| --- | --- | --- | --- | --- | --- | --- | --- | --- | --- | --- | --- | --- | --- | --- | --- |
|  | **Baseline spending** | **Optimised spending 100%** | **Optimised spending 150%** | **Baseline spending** | **Optimised spending 100%** | **Optimised spending 150%** | **Baseline spending** | **Optimised spending 100%** | **Optimised spending 150%** | **Baseline spending** | **Optimised spending 100%** | **Optimised spending 150%** | **Baseline spending** | **Optimised spending 100%** | **Optimised spending 150%** |
| BCG | $140,993 | $140,993 | $143,800 | $194,663 | $195,850 | $206,371 | $44,006 | $42,860 | $54,277 | $316,063 | $316,063 | $356,572 | $1,164,814 | $1,165,457 | $1,984,874 |
| TPT contacts | $1,071 | $1,372,877 | $1,383,386 | $40,117 | $314,406 | $314,646 | $9,165 | $90,723 | $90,822 | $749,472 | $7,539,995 | $20,343,575 | $605,464 | $4,257,872 | $4,574,429 |
| TPT PLHIV | $12,995 | $20,708 | $21,619 | $20,215 | $20,215 | $20,230 | $789 | $795 | $797 | $384,398 | $384,398 | $384,398 | $35,320 | $122,806 | $129,778 |
| Contact tracing | $10,954 | $389,332 | $387,522 | $136,865 | $125,292 | $182,334 | $177,498 | $316,279 | $317,182 | $15,398,122 | $7,699,061 | $7,699,061 | $461,082 | $1,089,909 | $1,119,618 |
| ACF prisoners | $10,817 | $5,408 | $5,516 | $12,404 | $6,202 | $33,049 | $0 | $0 | $0 | $405,141 | $202,570 | $408,541 | $293,036 | $667,025 | $1,423,122 |
| ACF people at higher risk, including PLHIV | $3,761 | $3,761 | $3,836 | $13,955 | $17,914 | $18,329 | $2,000,067 | $2,357,466 | $4,001,370 | $0 | $0 | $0 | $4,265,659 | $8,696,077 | $10,085,455 |
| ACF community/ mobile screening | $0 | $363,011 | $375,886 | $0 | $0 | $0 | $1,050,311 | $1,112,123 | $2,101,270 | $217,837 | $1,350,223 | $1,622,848 | $0 | $0 | $0 |
| ACF facility | $0 | $0 | $0 | $0 | $0 | $0 | $0 | $0 | $0 | $634,094 | $824,341 | $829,462 | $0 | $0 | $0 |
| Passive and other case finding | $129,837 | $102,645 | $134,076 | $1,371,541 | $1,395,437 | $2,290,422 | $349,555 | $332,709 | $387,005 | $0 | $0 | $0 | $14,857,743 | $14,958,855 | $15,665,507 |
| Mass screening & mandatory testing^a^ | $5,536,503 | $2,768,252 | $7,623,052 | $0 | $0 | $0 | $0 | $0 | $0 | $407,202 | $203,601 | $203,601 | $10,919,288 | $5,462,657 | $23,025,862 |
| DS-TB treatment | $2,875,938 | $3,353,420 | $4,179,785 | $6,140,472 | $6,275,603 | $8,986,961 | $6,181,472 | $5,541,511 | $7,684,202 | $6,383,922 | $6,990,663 | $7,266,359 | $28,417,223 | $25,945,027 | $30,870,112 |
| MDR-TB treatment (standard) | $1,805,552 | $902,776 | $920,745 | $5,590,834 | $2,795,417 | $2,797,447 | $1,814,406 | $923,727 | $939,788 | $1,297,876 | $648,938 | $689,671 | $16,989,749 | $10,624,453 | $14,475,492 |
| MDR-TB treatment (short) | $3,617,748 | $6,249,414 | $8,855,420 | $636,418 | $2,899,324 | $6,078,220 | $477,021 | $1,541,599 | $2,890,804 | $510,490 | $818,672 | $800,654 | $2,151,855 | $9,984,734 | $21,107,120 |
| XDR-TB treatment (standard) | $2,656,679 | $1,328,339 | $1,354,779 | $92,900 | $46,450 | $46,484 | $316,775 | $161,272 | $164,077 | $547,818 | $273,909 | $273,909 | $8,015,055 | $4,009,739 | $6,828,934 |
| XDR-TB treatment (short) | $396,177 | $198,089 | $409,114 | $0 | $158,275 | $401,083 | $0 | $0 | $0 | $0 | $0 | $0 | $780,715 | $1,972,392 | $2,145,203 |
| Total spending | $17,199,025 | $17,199,025 | $25,798,537 | $14,250,383 | $14,250,383 | $21,375,575 | $12,421,063 | $12,421,063 | $18,631,595 | $27,252,434 | $27,252,434 | $40,878,651 | $88,957,003 | $88,957,003 | $133,435,505 |

Notes: ACF, active case finding; BCG, Bacillus Calmette-Guérin; DS, drug susceptible; MDR, multi-drug resistant; PLHIV, people living with HIV; TB, tuberculosis; TPT, TB preventive treatment; XDR, extensively drug-resistant.

A, Mandatory testing considered in Uzbekistan only. Source: 2023 Optima TB country models

**Projected impact of baseline and optimised spending on TB-related deaths, new/relapse pulmonary TB infections and pulmonary TB incidence per 100,0000 population**

Table B. Projected impact of baseline and optimised spending on TB-related deaths, new/relapse pulmonary TB infections and pulmonary TB incidence per 100,0000 population

|  |  | **TB-related deaths** | | | **New/relapse pulmonary TB infections (n)** | | | **Pulmonary TB incidence per 100,000 population** | |
| --- | --- | --- | --- | --- | --- | --- | --- | --- | --- |
|  |  | **Cumulative 2024-2030, n** | **Difference from baseline, n [95% CI]** | **Difference from baseline, % [95% CI]** | **Cumulative 2024-2030, n** | **Difference from baseline, n [95% CI]** | **Difference from baseline, % [95% CI]** | **Projected rate in 2030** | **(IQR)** |
| Belarus | Baseline spending | 2128 |  |  | 15,941 |  |  | 23 | (21, 26) |
|  | Optimised spending 100% | 1783 | -346 [-540, -212] | -16% [-25%, -10%] | 14,221 | -1721 [-1967, -1283] | -11% [-12%, -8%] | 19 | (18, 21) |
|  | Optimised spending 150% | 1757 | -371 [-778, -189] | -17% [-37%, -9%] | 14,115 | -1826 [-2558, -1288] | -11% [-18%, -9%] | 19 | (18, 21) |
| Kyrgyz Republic | Baseline spending | 3160 |  |  | 51,960 |  |  | 97 | (86, 112) |
|  | Optimised spending 100% | 3145 | -15 [-91, 43] | 0% [-3%, 1%] | 51,641 | -319 [-446, -98] | -1% [-1%, 0%] | 95 | (85, 110) |
|  | Optimised spending 150% | 3002 | -158 [-394, -33] | -5% [-12%, -1%] | 51,394 | -566 [-969, -53] | -1% [-2%, 0%] | 92 | (83, 107) |
| Moldova | Baseline spending | 1393 |  |  | 12,028 |  |  | 48 | (38, 66) |
|  | Optimised spending 100% | 1225 | -168 [-451, -90] | -12% [-32%, -6%] | 10,770 | -1258 [-1957, -824] | -10% [-15%, -6%] | 39 | (30, 58) |
|  | Optimised spending 150% | 989 | -404 [-750, -136] | -29% [-54%, -10%] | 10,440 | -1587 [-2787, -859] | -13% [-23%, -7%] | 36 | (28, 52) |
| Tajikistan | Baseline spending | 6522 |  |  | 28,611 |  |  | 35 | (29, 43) |
|  | Optimised spending 100% | 5362 | -1160 [-1182, -299] | -18% [-18%, -5%] | 24,846 | -3765 [-4174, -1228] | -13% [-14%, -4%] | 27 | (22, 36) |
|  | Optimised spending 150% | 5092 | -1430 [-1528, -380] | -22% [-23%, -6%] | 22,078 | -6532 [-8543, -2449] | -23% [-34%, -10%] | 23 | (18, 30) |
| Uzbekistan | Baseline spending | 17,913 |  |  | 96,737 |  |  | 35 | (27, 58) |
|  | Optimised spending 100% | 15,030 | -2882 [-3222, 2419] | -16% [-18%, 14%] | 87,809 | -8928 [-9750, -3939] | -9% [-10%, -4%] | 29 | (23, 56) |
|  | Optimised spending 150% | 14,680 | -3232 [-4815, -2385] | -18% [-27%, -13%] | 87,418 | -9319 [-11613, -9143] | -10% [-12%, -9%] | 29 | (19, 51) |

Best estimates for cumulative outcomes are based on the modelled impact of changed spending for calibrated parameter sets. Interquartile range (IQR) of projections are used to represent uncertainty intervals as these most closely reflected WHO uncertainty intervals in 2022. The uncertainty range for difference from baseline is based on the 95% confidence interval (CI) from pairwise differences between parameter sets sampled with the same random seed, given changes in spending.
